# The DNA methylome of cervical cells can predict the presence of ovarian cancer

**DOI:** 10.1038/s41467-021-26615-y

**Published:** 2022-02-01

**Authors:** James E. Barrett, Allison Jones, Iona Evans, Daniel Reisel, Chiara Herzog, Kantaraja Chindera, Mark Kristiansen, Olivia C. Leavy, Ranjit Manchanda, Line Bjørge, Michal Zikan, David Cibula, Martin Widschwendter

**Affiliations:** 1 European Translational Oncology Prevention and Screening (EUTOPS) Institute, 6060 Hall in Tirol, Austria; 2 Research Institute for Biomedical Aging Research, Universität Innsbruck, 6020 Innsbruck, Austria; 3 Department of Women’s Cancer, UCL EGA Institute for Women’s Health, University College London, 74 Huntley Street, London, WC1E 6AU UK; 4 UCL Genomics, Zayed Centre for Research into Rare Disease in Children, University College London, London, WC1N 1DZ UK; 5 Department of Health Sciences, University of Leicester, Leicester, LE1 7RH UK; 6 Department of Non-communicable Disease Epidemiology, The London School of Hygiene and Tropical Medicine, London, WC1E 7HT UK; 7 Department of Gynaecological Oncology, Barts Health NHS Trust, Royal London Hospital, London, E1 1BB UK; 8 Centre for Prevention, Detection & Diagnosis, Wolfson Institute of Population Health, Queen Mary University of London, London, EC1M 6BQ UK; 9 Department of Health Services Research, The London School of Hygiene and Tropical Medicine, London, WC1E 7HT UK; 10 Department of Obstetrics and Gynaecology, Haukeland University Hospital, Bergen, Norway; 11 Centre for Cancer Biomarkers CCBIO, Department of Clinical Science, University of Bergen, Bergen, Norway; 12 Hospital Na Bulovce, Prague, Czech Republic; 13 Department of Obstetrics and Gynecology, General University Hospital in Prague, First Faculty of Medicine, Charles University, Prague, Czech Republic; 14 Department of Women’s and Children’s Health, Karolinska Institutet, Stockholm, Sweden

**Keywords:** Cancer screening, Ovarian cancer, Cancer epigenetics

## Abstract

The vast majority of epithelial ovarian cancer arises from tissues that are embryologically derived from the Müllerian Duct. Here, we demonstrate that a DNA methylation signature in easy-to-access Müllerian Duct-derived cervical cells from women with and without ovarian cancer (i.e. referred to as the Women’s risk IDentification for Ovarian Cancer index or WID-OC-index) is capable of identifying women with an ovarian cancer in the absence of tumour DNA with an AUC of 0.76 and women with an endometrial cancer with an AUC of 0.81. This and the observation that the cervical cell WID-OC-index mimics the epigenetic program of those cells at risk of becoming cancerous in *BRCA1/2* germline mutation carriers (i.e. mammary epithelium, fallopian tube fimbriae, prostate) further suggest that the epigenetic misprogramming of cervical cells is an indicator for cancer predisposition. This concept has the potential to advance the field of risk-stratified cancer screening and prevention.

## Abstract (web summary)

Most ovarian cancers originate from cells originally derived from Müllerian Duct cells. Here, the authors show that the methylation profile of Müllerian Duct cells isolated from cervical samples can predict whether a woman has cervical cancer.

## Introduction

Epithelial ovarian cancer is by far the most common cause of gynaecological cancer-associated death^1^. To address this, the biggest challenge faced has been to identify the group of women at the highest risk of developing this devastating disease for which measures to diagnose early and/or prevent the disease can be offered. Currently, the best performing model has used a combination of single nucleotide polymorphisms (SNPs) as well as a large number of epidemiological parameters and has achieved a Receiver Operating Characteristic (ROC) Area Under the Curve (AUC) of 0.66 in an internal validation set^2^. Recent data show that women with the highest and lowest 5% of the resulting polygenic risk score (PRS) have a 2.8% and a 0.9% risk of developing ovarian cancer until 80 years of age (the general risk for ovarian cancer in this population was 1.86%)^3^. Several other risk models using up to 214 combinations of various genetic and non-genetic risk/protective factors have been developed^4,5^. Hence, there is a clear need to improve upon the identification of women at risk of developing ovarian cancer in order to offer women at high risk those strategies which have been shown to detect ovarian cancer earlier^6,7^ or target preventative measures including removal of the fallopian tubes, the organ from which the majority of ovarian cancers originate^8^.

Recent evidence has shown that an assay which combines testing somatic mutations and aneuploidy in cervical brush samples is capable of identifying 33% of women presenting with ovarian cancer (although the majority of these women have advanced stage III/IV cancers)^9^. In this study, which aimed to detect tumour DNA, the average age of cases and controls was 58 and 34 years, respectively. The reported observations of a high allele frequency of pathogenic driver mutations in DNA from non-malignant normal uterine tissue with increasing age^10–13^ compromises the results of this test and makes it impossible to judge the true specificity of the test.

Epigenetic (i.e. DNA methylation, DNAme) changes have been identified in normal fimbriae from women with *BRCA1/2* germline mutations^14^ and could potentially serve as surrogate markers for both genetic and non-genetic factors including lifestyle, reproductive, and environmental exposures contributing to ovarian cancer development^15^. A number of proof of principle studies, so far performed exclusively in blood, have demonstrated that certain DNAme changes are associated with ovarian cancer predisposition^16–18^. Sample heterogeneity and the choice of surrogate tissue (i.e. tissue which is both easy to access and best reflects the tissue at risk) are among the most important factors impeding clinical implementation^19^.

The vast majority of epithelial ovarian cancers arise from structures derived from the Müllerian Duct and includes the fallopian tube and endosalpingiosis, endometriosis, and endocervicosis, representing non-neoplastic counterparts of serous, endometrioid/clear cell, and mucinous ovarian carcinomas, respectively^20^.

Here we assess whether DNAme profiles, derived from cervical smear samples that contain hormone sensitive epithelial cells capable of recording ovarian cancer-predisposing factors at the level of the epigenome^19^ and arising from the Müllerian Duct, are capable of identifying women with primary epithelial ovarian cancer. We take particular care to demonstrate that the DNAme profiles originate from the normal cervical sample and are not driven by tumour DNA.

We perform an epigenome-wide DNAme analysis in cervical smear samples from women who were subsequently diagnosed with ovarian cancer, and in matched controls (i.e. the Discovery Set), and establish/validate the WID-OC-index (Women’s risk IDentification for Ovarian Cancer index) which we then further validate in an independent set of cervical samples (i.e. External Validation Set). We establish that the WID-OC signature is not driven by tumour DNA, is high in healthy women with a *BRCA1* mutation as well as in women with poor prognostic breast cancer and furthermore has a very high sensitivity and specificity for identifying women with endometrial cancer, which like ovarian cancer also arises from Müllerian Duct epithelial cells. In addition, we assess the WID-OC-index in normal fimbrial and high-grade serous tissue and cell lines, and in a large range of tissue samples, and find that the WID-OC-index is significantly associated with those tissues that show a high rate of *BRCA-1/2* germline-mediated cancer formation.

## Results

For the Discovery Set (Supplementary Fig. 1), we collected samples from 242 women with ovarian cancers from 15 European centres before a definitive histological diagnosis was undertaken (either during surgery or via a percutaneous biopsy) and 869 women without a cancer (593 from the general population and 276 from women attending hospital for benign women-specific conditions) (Supplementary Table 1; samples from a greater proportion of younger women were deliberately used in the discovery set in order to develop a risk predictor which was also applicable to younger women; the external validation set was composed of age-matched cases and controls). Epigenome-wide DNAme was analysed using an Illumina Infinium EPIC bead chip array that encompasses over 850,000 CpG sites^21^.

### Sample heterogeneity and differential methylation

**Fig. 1:**
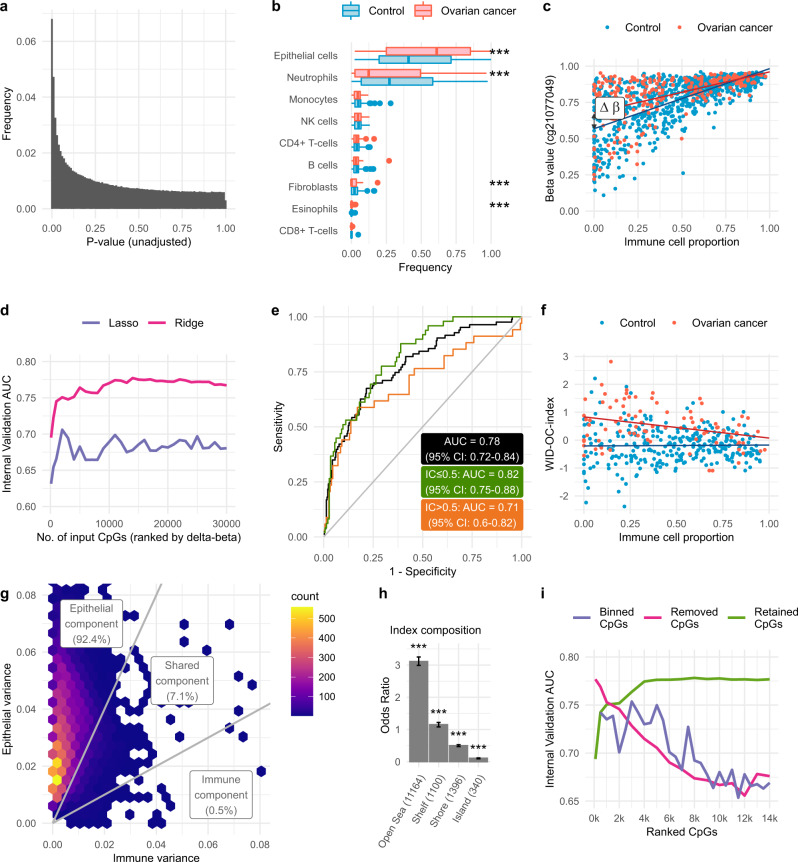
Sample heterogeneity, differential methylation, and development of discriminatory index. **a** Distribution of *p*-values obtained by comparing cases and controls at each CpG site and after controlling for immune cell proportion and age. **b** The distribution of different cell types in the discovery dataset inferred using the EpiDISH algorithm. *p*-values were computed using a two-tailed Mann–Whitney test. For indicated significant differences, exact *p*-values = 0.00014 (epithelial), <0.0001 (neutrophil), <0.0001 (fibroblast), <0.0001 (eosinophil). The centre line of each box corresponds to the median. The lower and upper hinges correspond to the first and third quartiles. The upper whisker extends from the hinge to the largest value no further than 1.5 * IQR from the hinge (where IQR is the inter-quartile range, or distance between the first and third quartiles). The lower whisker extends from the hinge to the smallest value at most 1.5 * IQR of the hinge. Data beyond the end of the whiskers are plotted individually. **c** An example of a CpG with epithelial specific differential methylation. **d** Area under the curve (AUC) values in the internal validation set as a function of the number of CpGs used to train the classifier. **e** ROC curves of the WID-OC-index in the internal validation set for samples with an immune cell (IC) proportion ≤0.5 and >0.5. **f** Distribution of the WID-OC-index with respect to immune cell proportion in the internal validation set. **g** Distribution of the estimated variance in epithelial and immune cells across all CpGs used in the WID-OC- index. **h** Odds ratios corresponding to the four genomic regions when comparing the CpGs used in the WID-OC-index to the overall EPIC array. Error bars correspond to 95% confidence intervals computed using the median-unbiased estimation method. For indicated significant differences, all exact *p* values <0.0001. **i** AUC values in the internal validation set after training classifiers on different subsets of the CpGs used in the WID-OC-index. The top n CpGs were either retained or removed. CpGs were also split into separate bins of size 500. Source data are provided as a Source Data file.

We assessed the number of CpGs, which were significantly differentially methylated between cancer cases and controls (Fig. 1a); after Bonferroni multiple test adjustment, 91 CpGs showed significantly differential methylation (Supplementary Table 2). Previously, we found that methylation differences may vary due to immune cell-type composition in cases compared to controls^22^. Hence, we assessed the level of cell type heterogeneity in each cervical smear sample using HEpiDISH^23^, an algorithm that infers the relative proportion of epithelial cells, fibroblasts, and seven subtypes of immune cells (IC) in each sample. The cell-type distributions were broadly similar between cancer cases and controls although there was a significantly greater proportion of epithelial cells in cancer cases (Fig. 1b; this remained significant after adjusting for age and menopausal status). A similar trend was observed in the external validation dataset but was not significant (Supplementary Fig. 2a).

Identification of CpGs with differential methylation between cases and controls was hampered by contaminating ICs, since any differential methylation in epithelial cells was greatly diminished in samples with high IC. In order to infer which CpGs may contain a potential discriminatory signal we developed a statistical protocol to estimate the delta-beta (i.e. difference in mean proportion of methylated cells) between cases and controls in epithelial and immune cells. We linearly regressed beta values on IC fraction in both cases and controls separately. The difference between the two points where these lines intercept the *y*-axis at IC = 0 gives an estimate of the delta-beta between cases and controls in pure epithelial cells (Fig. 1c). Conversely, the difference between intercept points at the IC = 1 axis gives a delta-beta estimate in immune cells.

### Development of discriminatory index

In order to derive a diagnostic methylation signature, i.e. the WID-OC-index, we used ridge and lasso regression to classify individuals as cases or controls. Classifiers were trained on two thirds of the discovery dataset (572 cancer-free controls, 159 ovarian cancer cases) and the remaining one third was used as an internal validation set (297 controls, 83 cases) with the intention of evaluating their performance as a function of the number of CpGs used to construct the index (Supplementary Fig. 1). The area under the receiver operator characteristic curve (AUC) was used as a measure of predictive performance. CpGs were ranked according to their epithelial delta-beta.

Predictive performance was evaluated as a function of the number of CpGs used to train the classifier using the internal validation dataset and an optimal performance of 0.78 (95% CI: 0.72–0.84) was achieved using 14,000 CpGs (Supplementary Data 1) with ridge regression (Fig. 1d; i.e. the WID-OC-index is defined as a linear combination of these 14,000 beta values). In samples with an immune cell proportion ≤0.5 the AUC was 0.82 (Fig. 1e; 95% CI: 0.75–0.88), and in those with a proportion >0.5 the AUC was 0.71 (95% CI: 0.60–0.82). The WID-OC-index was not associated with IC fraction in controls (Fig. 1f, linear regression coefficients of 0.03, *p* = 0.81), but a strong negative association was observed in cancer cases (linear regression coefficient of −0.76, *p*-value = 0.005). Classifiers were also developed after ranking CpGs according to immune delta-betas and a combined ranking based on both epithelial and immune delta-betas but these approaches offered inferior performance.

We developed a statistical model to infer the variance in epithelial and immune cells at each of the 14,000 CpG sites used in the WID-OC-index, and classified each CpG as “epithelial” (92.4%), “shared” (7.1%), or “immune” (0.5%) as shown in Fig. 1g. These findings suggest that the discriminatory signal originates primarily in epithelial cells and the discriminatory power is diminished in samples with higher levels of immune cells.

We found that the index was highly depleted of CpG islands and enriched for Open Sea regions (Fig. 1h). Ridge regression combines information from all input CpGs in contrast to lasso regression, which typically selects a small subset of inputs. Ridge regression offered consistently superior performance suggesting that the discriminatory signal is most robustly extracted by combining a large number of comparatively weak signals from multiple CpG sites. We ranked the 14,000 CpGs used to define the WID-OC-index according to the absolute value of the regression coefficients from the ridge model. In order to assess how informative the top CpG sites are we trained sub-classifiers on the top *n* sites (Fig. 1i). We observed that AUCs of 0.74 and 0.76 could be achieved with the top 500 and 3,000 CpGs, respectively, indicating that these subsets are particularly informative. We also trained sub-classifiers after removing the top *n* CpGs, and on subsets of 500 CpGs after partitioning the ranked list into bins of size 500. In both cases, we found that a substantial predictive signal is present. These observations suggest that the predictive signal is widely distributed among Open Sea CpGs with a high degree of redundancy.

### External validation

**Fig. 2:**
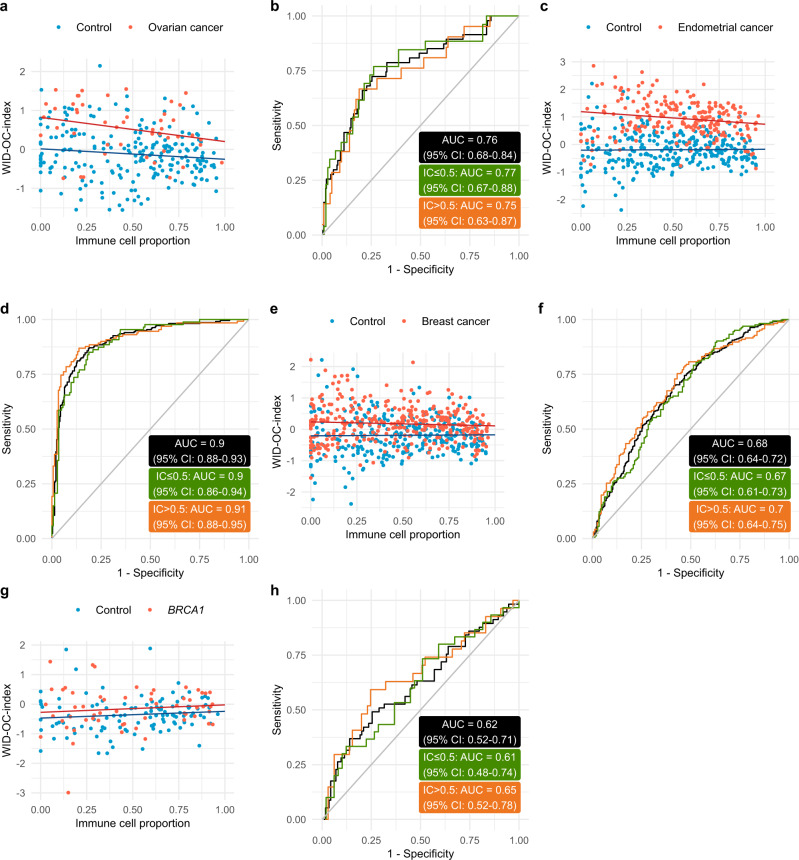
External validation. **a** The WID-OC-index versus immune cell proportion in an independent external validation set. **b** ROC curve from the external validation set. **c** The WID-OC-index versus immune cell proportion in a separate cohort of endometrial cancer samples and the same control samples from the internal validation set. **d** ROC curve from the endometrial cancer dataset. **e** The WID- OC-index versus immune cell proportion in a separate cohort of breast cancer samples and the same control samples from the internal validation set. **f** ROC curve from the breast cancer dataset. **g** The WID-OC-index versus immune cell proportion in an independent cohort of *BRCA1* mutation carriers. **h** ROC curve from the *BRCA1* dataset. Source data are provided as a Source Data file.

A separate independent external validation dataset consisting of 47 ovarian cancer cases and 225 controls was used to validate the index performance (Supplementary Fig. 1). The WID-OC-index was computed for each woman (Fig. 2a) resulting in an overall AUC of 0.76 (95% CI: 0.68–0.84), with the AUC being 0.77 (95% CI: 0.67–0.88) and 0.75 (95% CI: 0.63–0.87) for IC ≤ 0.5 and >0.5 samples, respectively (Fig. 2b).

The vast majority of ovarian cancers arise from the fallopian tube—a part of the Müllerian Duct system. In order to assess whether the WID-OC-index reflects cancer predisposition of other parts of the Müllerian Duct such as the endometrium, we assessed its performance to discriminate women with and without endometrial cancer. Analysing 217 cervical samples from women with endometrial cancer, and the 297 control samples from the ovarian cancer internal validation set, we obtained an AUC of 0.90 (95% CI: 0.88–0.93; Fig. 2c, d).

To further assess the risk predictive nature of the WID-OC-index we analysed 329 cervical samples from women with primary breast cancers with poor prognosis features (defined as >2 cm cancers and/or lymph-node positive and/or hormone-receptor negative and/or grade 3) and the 297 control samples from the ovarian cancer internal validation set. We obtained an AUC of 0.68 (95% CI: 0.64–0.72; Fig. 2e, f). The ability of the WID-OC-index to discriminate between healthy controls and women with an anatomically distant cancer supports the interpretation that the index reflects cancer predisposition rather than a diagnostic signal based on tumour DNA.

In order to assess whether the WID-OC-index is also informative in healthy women who are known to have an extremely high probability of developing ovarian cancer in the future, we analysed a separate dataset consisting of cervical smear samples from 57 healthy *BRCA1* mutation carriers (who have an up to 40 fold increased cancer risk^24^) and 114 *BRCA1/2* wild-type controls (Fig. 2g). We observed an AUC of 0.62 (95% CI: 0.52–0.71; Fig. 2h) overall with an AUC of 0.61 (95% CI: 0.48–0.74) and 0.65 (95% CI: 0.52–0.78) for IC ≤ 0.5 and >0.5 samples, respectively. We also analysed 53 women with a *BRCA2* mutation and found that the discriminatory performance was poorer (0.54; 95% CI: 0.45–0.64; Supplementary Fig. 3).

For each of our validation datasets we computed odds ratios corresponding to quartiles defined on the internal validation dataset (Table 1). We also computed the specificities and sensitivities in different age groups at different cutoffs (Supplementary Table 3). The cell-type composition of the four validation datasets described was broadly similar to the discovery dataset used to develop the index and did not show any significant differences between cases and controls (Supplementary Fig. 2).

**Table 1:** Odds ratios corresponding to quartiles defined using the internal validation ovarian cancer dataset.

Quartile	Control (*N* = 297)	Internal validation ovarian cancer (*N* = 83)	Unadjusted OR (95% CI)	Adjusted OR (95% CI)
**(−2.38, −0.57)**	75	3	1.00 (reference)	1.00 (reference)
**(−0.57, −0.21)**	74	10	3.25 (0.93,15.68)	2.38 (0.56, 12.99)
**(−0.21, 0.17)**	74	13	4.20 (1.27,19.79)	3.65 (0.87, 19.42)
**(0.17, 2.21)**	74	57	18.20 (6.33,79.95)	10.26 (2.89, 49.1)

**Quartile**	**Control (*N*** **=** **225)**	**External validation ovarian cancer (*N*** **=** **47)**	**Unadjusted OR (95% CI)**	**Adjusted OR (95% CI)**
**(−2.38, −0.57)**	59	2	1.00 (reference)	1.00 (reference)
**(−0.57, −0.21)**	57	6	2.94 (0.62,22.94)	4.88 (0.85, 41.76)
**(−0.21, 0.17)**	49	6	3.42 (0.72,26.71)	4.57 (0.77, 40.12)
**(0.17, 2.21)**	60	33	14.99 (4.26,103.18)	26.25 (5.89, 194.92)

**Quartile**	**Control (*N*** **=** **297)**	**Endometrial cancer (*N*** **=** **217)**	**Unadjusted OR (95% CI)**	**Adjusted OR (95% CI)**
**(−2.38, −0.57)**	75	4	1.00 (reference)	1.00 (reference)
**(−0.57, −0.21)**	74	6	1.50 (0.4,6.32)	0.72 (0.17, 3.26)
**(−0.21, 0.17)**	74	15	3.68 (1.25,13.77)	0.92 (0.24, 3.92)
**(0.17, 2.21)**	74	192	46.44 (18.41,159.14)	11.20 (3.91, 40.51)

**Quartile**	**Control (*N*** **= 297)**	**Breast cancer (*N = 329)***	**Unadjusted OR (95% CI)**	**Adjusted OR (95% CI)**
**(−2.38, −0.57)**	75	28	1.00 (reference)	1.00 (reference)
**(−0.57, −0.21)**	74	49	1.77 (1.01,3.14)	1.50 (0.83, 2.76)
**(−0.21, 0.17)**	74	91	3.27 (1.94,5.64)	2.30 (1.29, 4.14)
**(0.17, 2.21)**	74	161	5.78 (3.49,9.8)	5.27 (2.91, 9.78)

**Quartile**	**Control (*N*** **= 114)**	***BRCA1*** **cases (*N*** **= 87)**	**Unadjusted OR (95% CI)**	**Adjusted OR (95% CI)**
**(−2.38, −0.57)**	41	12	1.00 (reference)	1.00 (reference)
**(−0.57, −0.21)**	26	15	1.95 (0.79,4.95)	1.66 (0.64, 4.31)
**(−0.21, 0.17)**	31	12	1.32 (0.51,3.39)	1.20 (0.42, 3.4)
**(0.17, 2.21)**	16	18	3.76 (1.49,9.88)	2.59 (0.84, 8.08)

Adjustment was based on a logistic regression model with age, menopausal status, age at menarche, number of first degree relatives with ovarian cancer, and BMI included as covariates for the ovarian cancer datasets. For endometrial cancers and the *BRCA1* dataset age and menopause were included as covariates. For the endometrial cancers adjusted estimates are unavailable as the logistic regression model failed to converge. In addition, it was assumed that there was 1 cancer case in the first quartile in order to estimate ORs for the remaining quartiles.

### Association with epidemiological, clinical, and technical factors

**Fig. 3:**
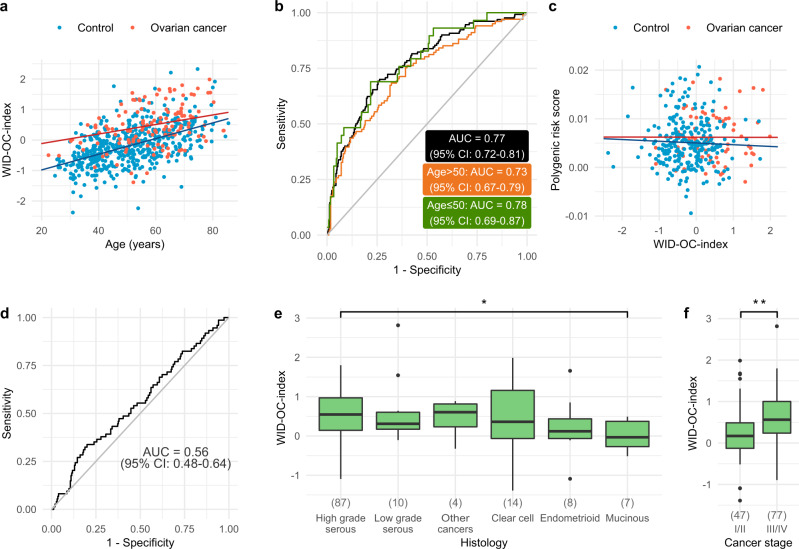
Association with epidemiological, clinical, and technical factors. **a** The WID-OC-index versus age in control samples from the internal and external validation datasets. **b** ROC curves for women above and below 50 years of age. **c** The WID-OC-index versus a 28 SNP ovarian cancer polygenic risk score (PRS) in the internal validation dataset. **d** ROC curve corresponding to the PRS score. **e** The distribution of the WID-OC-index across different histological subtypes (endometrioid borderline, mucinous-clear cell cancer, carcinosarcoma, and serous cancer with no information on grade have been classified as “other cancers”). *p*-values were computed using a two-tailed Mann–Whitney test. For indicated significant difference, exact *p*-value = 0.013. **f** The distribution of the WID-OC-index across different cancer stages. *p*-values were computed using a two-tailed Mann–Whitney test. For indicated significant difference, exact *p* value = 0.0023. For box plots in **e** and **f**, the centre line of each box corresponds to the median. The lower and upper hinges correspond to the first and third quartiles. The upper whisker extends from the hinge to the largest value no further than 1.5 * IQR from the hinge (where IQR is the inter-quartile range). The lower whisker extends to the smallest value at most 1.5 * IQR of the hinge. Data beyond the end of the whiskers are plotted individually. Source data are provided as a Source Data file.

We investigated the relationship between the WID-OC-index and various epidemiological and clinical variables. A statistically significant association was found between the WID-OC-index and age in controls (correlation = 0.52, *p* = 10^−37^; Fig. 3a), which resulted in a slightly better performance of the index in younger women (Fig. 3b). The Illumina 650k Infinium Global Screening Array was used to genotype matched blood samples from a subset of 74 cases and 255 controls in our internal validation dataset. We computed a polygenic risk score (PRS; described in methods; Supplementary Table 4) for ovarian cancer prediction. We found a correlation close to zero (−0.04, *p* = 0.48) between the PRS and the WID-OC-index (Fig. 3c) and the PRS was not predictive in this set (Fig. 3d). We compared the different cancer histologies and interestingly found a significant and progressive decrease of the WID-OC-index according to the longitudinal anatomy of the Müllerian Duct [i.e. fallopian tube (serous), endometrium (clear cell and endometrioid), and endocervix (mucinous)] (Fig. 3e). The WID-OC-index was significantly higher in stage III/IV cancers compared to stage I/II cancers (Fig. 3f). No significant association in controls was found between the WID-OC-index and family history (Supplementary Fig. 4a), age at menarche (Supplementary Fig. 4b), oral contraceptive pill use (Supplementary Fig. 4c), or ethnicity (Supplementary Fig. 4d). We observed a trend of increasing WID-OC-index values with respect to age at menopause (Supplementary Fig. 4e; linear regression *p*-value = 0.01), and parity in post-menopausal women (Supplementary Fig. 4f; linear regression not significant).

We investigated whether any association existed between the WID-OC-index and various technical parameters including date of sample processing, plate number (samples were processed on 96 sample plates), and sentrix position but no significant associations were found. We compared the 593 control samples from healthy volunteers to 276 control samples taken from women presenting with benign women-specific conditions but did not find any significant differences (Supplementary Fig. 5a). We also observed no significant dependence on the time from sample collection to DNA extraction (Supplementary Fig. 5b). The performance of the WID-OC-index to discriminate between healthy controls and women with ovarian, endometrial, or breast cancer was highly consistent across different study centres (Fig. S5c–e).

CA-125 data on 48 samples (40 cancers and 8 controls) from the internal and external dataset had a non-significant correlation of 0.14 (*p* = 0.35) with the WID-OC-index.

### Inferred proportion of tumour DNA

**Fig. 4:**
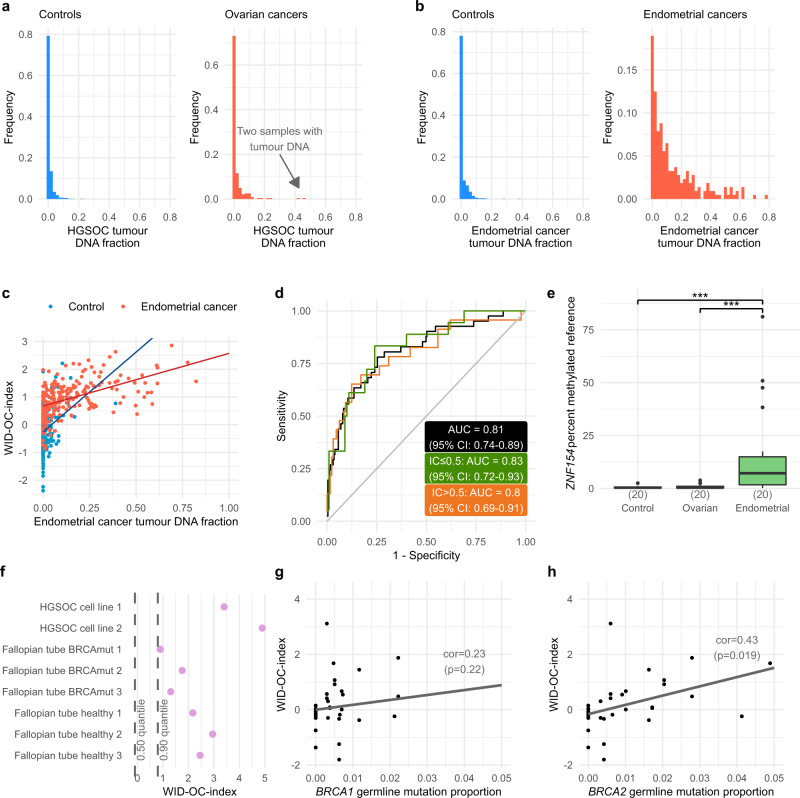
Inferred proportion of tumour DNA and functional assessment of the WID-OC-index. The estimated proportion of tumour DNA in each cervical smear sample as estimated using the EpiDISH algorithm for controls and **a** ovarian cancers and **b** endometrial cancers. **c** Distribution of the WID-OC-index with respect to tumour DNA fraction in controls and endometrial cancers. **d** ROC curve for samples with tumour DNA < 1% in the endometrial cancer set. **e** Results from real-time PCR to detect *ZNF154*, a pan-cancer marker primarily discovered in ovarian cancer. *p*-values were computed using a two-tailed Mann–Whitney test. For indicated significant differences, all exact *p* values < 0.001. The centre line of each box corresponds to the median. The lower and upper hinges correspond to the first and third quartiles. The upper whisker extends from the hinge to the largest value no further than 1.5 * IQR from the hinge (where IQR is the inter-quartile range). The lower whisker extends to the smallest value at most 1.5 * IQR of the hinge. Data beyond the end of the whiskers are plotted individually. **f** The WID-OC-index evaluated in eight different cell lines. **g**, **h** shows a subset of ENCODE tissue samples. The germline mutation proportion refers to the proportion of cancers in each tissue type that have a *BRCA1* and *BRCA2* mutation. *p*-values were computed using a two-tailed correlation test. Source data are provided as a Source Data file.

Due to the anatomical proximity between the cancer (i.e. ovary/fallopian tube) and the area from which the sample was taken (i.e. cervix), we investigated whether the signal, which discriminates between a case and a control, is driven by tumour DNA draining from the peritoneal cavity via the fallopian tube and the uterus to the cervix or whether the signal is a generic risk signal retained in cervical epithelial cells. We used 11 epithelial, 7 fibroblast, 42 immune cell, and 11 high-grade serous ovarian carcinoma (HGSOC) cell line samples (Supplementary Table 5) in order to develop a new reference panel for use with the EpiDISH algorithm (see Methods). For each sample, we obtained estimates of the proportion of DNA from each of the four cell types. We observed that the proportion of tumour DNA in both cases and controls is close to zero, with the exception of two cases that were composed of approximately 50% tumour DNA (Fig. 4a).

We used 9 endometrial cancer samples to derive an additional EpiDISH reference panel to infer the proportion of tumour DNA in samples from women with endometrial cancer. We found a substantial level of contaminating tumour DNA with 43% of samples from women with cancer containing >10% tumour DNA (Fig. 4b). Importantly, we found that the WID-OC-index discriminated between cases and controls in samples with no tumour DNA (Fig. 4c). In 229 controls and 41 cases with a tumour DNA proportion <1% the AUC was 0.81 (95% CI: 0.74–0.89; Fig. 4d).

In order to assess further the absence of tumour DNA in the cervical smear samples we used MethyLight, a real time PCR-based method, to amplify methylated *ZNF154*, a pan-cancer marker primarily discovered in ovarian cancer^25,26^. We detected a strong signal in cervical smear samples from 20 endometrial cancer cases but not from 20 ovarian cancer patients or 20 cancer-free women (Fig. 4e), providing further evidence that the signal in cancer patients is not driven by tumour DNA.

We performed a copy number variation (CNV) analysis of the cervical samples based on EPIC array data using the *conumee* R package^27^. We found that samples with a high inferred tumour DNA had a CNV profile very similar to the CNV profiles of HGSOC samples (Supplementary Fig. 6a, b). In contrast, samples with no inferred tumour DNA had completely normal CNV profiles (Supplementary Fig. 6c). The median and standard deviation of the estimated copy number per genomic bin in cervical samples was close to zero, consistent with our estimation that almost all of these samples contain no tumour DNA (Supplementary Fig. 6d, e). The standard deviation of the estimated copy number correlated strongly with estimated tumour DNA fraction (Supplementary Fig. 6f). These findings suggest that the estimated genomic copy number profiles are highly consistent with the inferred tumour DNA proportion.

Finally, we performed an in silico experiment to estimate how much contaminating tumour DNA would be required to explain the observed difference between cases and controls in the internal validation dataset (the mean value of the index in cases was 1.25 standard deviations higher than the mean in controls). We numerically simulated a mixture of beta values from a HGSOC cell line sample and the 144 control samples with an immune cell proportion <0.5 in the internal validation set. We estimated that we would need approximately 25% tumour DNA in order to observe a comparable difference to what was in fact observed (Supplementary Fig. 7a). Taken together these findings strongly suggest that the WID-OC-index does not depend on tumour by-products, and is instead based on the local cervical epigenome. This is also consistent with the detection of women with breast cancer, where anatomical distance precludes the presence of tumour material.

### Functional assessment of the WID-OC-index

In order to evaluate further the nature and meaning of the DNAme signature which forms the WID-OC-index we calculated the index in the fimbriae of the fallopian tube, the organ from which the vast majority of ovarian cancers arise and which originates from the Müllerian Duct, the embryological structure which also gives rise to the cervix. Since the fimbrial tissue contains a heterogenous set of various cells, we isolated and cultured pure fimbrial cells (without any modification, see Methods) from surgical specimens. Interestingly, the WID-OC-index was comparatively high in the normal fimbrial cells (Fig. 4f). Two cancer cell lines had higher WID-OC-index values compared to both the cervix-based WID-OC-index and the fimbrial cells. The observation that the discriminatory DNAme signature observed in the cervixes of ovarian cancer patients is more pronounced in the fallopian tube is suggestive of an epigenetic field defect in which DNAme patterns in the cervix resemble the epigenome of the cell of origin, the fallopian tube.

In order to assess additionally whether the WID-OC-index is reflective of a cell-specific program we analysed all ENCODE^28^ samples for which EPIC array data were available (Supplementary Table 6). We ranked and plotted the WID-OC-index in all primary cell samples and in vitro differentiated cell samples (Supplementary Fig. 8). We observed that those tissues at the highest risk of becoming cancerous in *BRCA* carriers—such as fallopian tube, breast, pancreas, and prostate—had the highest WID-OC-index. In order to quantify this observation, we correlated the proportion of cancers per organ which arise in *BRCA1* and *BRCA2* mutation carriers^29^ with the WID-OC-index of the respective normal tissue and found correlations of 0.23 (*p* = 0.22) and 0.43 (*p* = 0.019) in *BRCA1*- and *BRCA2-*driven cancers, respectively (Fig. 4g, h).

Finally, a gene set enrichment analysis was performed using the Broad Institute’s Molecular Signatures database but no significantly enriched pathways were detected (Supplementary Table 7).

## Discussion

To date, the best performing ovarian cancer risk prediction model which incorporates 17 established epidemiologic risk factors and 17 genome-wide significant SNPs using data from 11 case–control studies provided an AUC of 0.66. Here we demonstrate that the WID-OC-index—an index that is purely based on a DNAme signature in cervical smear samples—can predict ovarian cancer risk with an AUC of 0.76. We did not observe a striking association between the WID-OC-index and any of the known epidemiological risk factors for ovarian cancer (apart from age and a *BRCA1* germline mutation), and neither did we find any evidence that the WID-OC-index is triggered by tumour DNA draining from the peritoneal cavity via the uterine cavity and detected by the cervical smear. The absence of detectable tumour DNA, in conjunction with our in silico simulations which suggest that approximately 25% tumour DNA contamination would be required to account for the magnitude of the signal we have observed, imply that the index is based on cervical epigenetic profiles rather than tumour by-products.

Therefore, we speculate that the striking ovarian cancer risk reflected by the WID-OC-index is due to an epigenetic Müllerian Duct differentiation defect assessed at the level of the uterine cervix (a part of the Müllerian Duct) using a DNAme signature. Several lines of evidence support this proposal: (i) the WID-OC-index is high in fallopian tube fimbrial cells and reflective of organs that are at high risk of a *BRCA*-mediated cancer; (ii) the index also identifies women with other cancers despite the absence of tumour DNA arising from the Müllerian Duct (i.e. endometrial cancer); (iii) HOXA 9, 10, and 11 genes regulate the differentiation of the Müllerian Duct into fallopian tube, uterus, and cervix, respectively, and given that serous, clear cell/endometrioid, and mucinous cancers express these genes differentially, may reflect their origin^30^. The fact that we observe a decrease of the WID-OC-index from serous to clear cell/endometrioid to mucinous cancers again suggests a “shift” of epigenetic differentiation (i.e. differentiation of fallopian tube reflected in cervical epithelial cells) predisposes individuals to ovarian cancer formation; (iv) given that the index is more discriminatory in samples with a high epithelial cell content and that the index almost exclusively consists of CpGs, which are highly variable in epithelial but not in immune cells, attests to this. The fact that the WID-OC-index also identifies women with breast cancer supports the clinically well-known link between breast and ovarian cancer predisposition^31^ and again testifies that the index derived in cervical liquid-based cytology samples is not driven by tumour DNA.

Whether the WID-OC-index can identify women at risk of developing future ovarian cancer will have to be assessed by studying population-based cervical samples (from within a cervical screening cohort) and linked to cancer-registries; establishing such a biobank of samples stored as a pellet at −80 °C and waiting until a sufficiently large number of donors have developed ovarian cancer will take some time.

The WID-OC-index requires assessment of a relatively large number of CpGs. Whether the number of regions required to assess the WID-OC-index by assessing CpGs within the neighbourhood of the index CpGs (i.e. using target sequencing or real-time PCR) is able to achieve a similar performance will need to be studied.

Currently, 75% of ovarian cancers are picked up at an advanced stage where the tumour has spread within the entire abdominal cavity and beyond^1^. The WID-OC test identifies 71.4% and 54.5% of <50 and ≥50 year old ovarian cancer patients with a specificity of 75%. Further clinical studies will demonstrate whether women identified to have a high WID-OC-index (e.g. highest 25%) would benefit from regular screening using cell-free DNA and/or advanced imaging technologies.

Overall, we provide considerable evidence that an epigenetic differentiation defect in these easy-to-access epithelial samples is strongly associated with cancer risk.

## Methods

### Study design and epidemiological data acquisition

The study was conducted as part of a multi-centre study involving several recruitment sites in five European countries (i.e. the UK, Czech Republic, Italy, Norway, and Germany). Women over the age of 18 were eligible to participate in the study who had not undergone a previous hysterectomy, had not received treatment (within 2 years of recruitment) for a non-gynaecological cancer, were not pregnant or menstruating at the time of recruitment, and had not undergone a cervical smear in the last 12 weeks. Prior to taking part, each prospective study volunteer was given a Participant Information Sheet as well as a Consent Form and the rationale for the study was explained. Additional resources, including an explanatory video and further online resources, were also made available. Women diagnosed with ovarian or endometrial cancer (case) or a non-malignant benign gynaecological condition (control) were approached during outpatient hospital clinics, while women recruited with a documented *BRCA1* or *BRCA2* mutation or as healthy volunteers from the general population (control) were approached via outreach campaigns, public engagement, and as part of cervical screening programmes. After signing an informed consent, participants completed an epidemiological questionnaire as well as a feedback form after their participation. The study itself is a sub-study of the FORECEE (4 C) Programme, which has ethical approval from the UK Health Research Authority (REC 14/LO/1633).

The epidemiological survey was administered via the Qualtrics web-based survey application on dedicated iPads. The survey contained questions relating to current and historical health habits, relevant risk factors, as well as obtaining a thorough medical and obstetric history. Cervical samples were collected at appropriate clinical venues by trained staff and the cervical smears were carried out by a small group of research midwives or physicians with a view to establishing standard practice.

Biological samples and survey data were pseudo-anonymised using a participant study number. Each recruitment site maintained a securely stored file linking personal identifiers to the study number. Following sample taking, an email survey was sent to each participant, enabling them to feedback with respect to the recruitment process. Women with a current diagnosis of (a) primary malignant ovarian cancer of high grade serous, endometrioid, mucinous or clear cell morphology or (b) endometrial cancer with poor prognostic features (endometrioid, serous or clear cell morphology of Grade III and/or stage > IB) and recruited prior to receiving any systemic chemotherapy treatment or surgery or radiotherapy were eligible as ovarian or endometrial cancer cases. Cancer histological data was collected post-recruitment either by clinicians directly involved in the diagnosis/treatment of the cancer cases or by a nominated data manager with access to the in-house hospital systems.

### Cervical smear sample collection

Cervical smears were taken at collaborating hospitals and recruitment centres using the ThinPrep system (Hologic Inc., cat #70098-002). Cervical cells were sampled from the cervix using a cervix brush (Rovers Medical Devices, cat #70671-001) which was rotated 5 times through 360 degrees whilst in contact with the cervix to maximise cell sampling. The brush was removed from the vagina and immersed in a ThinPrep vial containing Preservecyt fluid and then pushed against the bottom of the vial 10 times in order to facilitate release of the cells from the brush into the solution. The sample vial was sealed and stored locally at room temperature.

### Primary fallopian tube secretory epithelial cell culture

Patients undergoing a salpingectomy at University College London Hospitals (UCLH), provided a written informed consent in order to donate fallopian tube tissue surplus to diagnostic requirements following UCL ethical guidelines (samples were collected under the NRES Committee London—Surrey Borders Research Ethics Committee approval; 14/LO/1633). Fimbrial fallopian tube secretory epithelial cells were isolated and cultured. Briefly, fimbrial tissues were carefully excised by an experienced pathologist, macerated, and digested in a dissociation medium (0.05% collagenase and 0.01% DNase in DMEM) for 48 h at 4 °C. Cells were harvested by centrifugation, resuspended in DMEM/F-12 supplemented with 2% Ultroser G (Pall Corporation, France) and 1% penicillin-streptomycin, and transferred into a tissue culture flask. Cells were phenotyped: firstly by determining mRNA expression of PAX8 (Müllerian marker) and Cytokeratin 7 (CK7, epithelial marker) using quantitative PCR; and secondly by immunofluorescent staining. All experiments were performed before the cells started to senesce and all FT cells were used without modification (such as hTERT or SV40T antigen immortalisation) in order to enhance self-renewal.

### Sample processing and DNA extraction

When preparing for sample storage in the laboratory, cervical smear samples were poured into 50 ml falcon tubes and left to sediment at room temperature for 2 h. 1 mL wide bore tips were then used to transfer the enriched cellular sediment into a 2 mL vial. The cervical sediments were washed twice with PBS, lysed, and stored temporarily at −20 °C ahead of extraction. For ovarian tissues, DNA was extracted from 30 mg of tissue using the AllPrep DNA/RNA Mini Kit (#80204, Qiagen Ltd), following the manufacturer’s protocol. DNA concentration and quality absorbance ratios were measured using Nanodrop-8000, Thermoscientific Inc. Extracted DNA was stored at −80 °C until further analysis.

### DNA methylation array analysis

Cervical, and fallopian tube and ovarian cancer cell line DNA was normalised to 25 ng/µl and 500 ng total DNA was bisulfite modified using the EZ-96 DNA Methylation-Lightning kit (Zymo Research Corp, cat #D5047) on the Hamilton Star Liquid handling platform. 8 µl of modified DNA was subjected to methylation analysis on the Illumina InfiniumMethylation EPIC BeadChip (Illumina, CA, USA) at UCL Genomics according to the manufacturer’s standard protocol.

### Real-time PCR analysis

MethyLight, a quantitative PCR analysis specific to bisulfite converted DNA, was performed on cervical smear samples from 20 endometrial cancer patients, 20 ovarian cancer patients, and 20 controls collected as part of the FORECEE project. Primers and probes specific to *ZNF154* were used (Supplementary Table 8). Ct values of the target reaction were normalised for DNA concentration using a reference gene reaction against *COL2A1* (Supplementary Table 8). Specificity of the reactions for methylated DNA was confirmed separately using SssI‐treated fully methylated human white blood cell DNA. The percentage of fully methylated molecules at a specific locus was calculated by dividing the *ZNF154:COL2A1* ratio of a sample by the *ZNF154:COL2A1* ratio of the SssI‐treated human white blood cell DNA and multiplied by 100. Results are expressed as ‘PMR’ (percentage of methylated ref. ^32^).

### Processing of the DNA methylation data

All methylation microarray data were processed through the same standardised pipeline. Raw data was loaded using the R package *minfi*. Any samples with median methylated and unmethylated intensities <9.5 were removed. Any probes with a detection *p*-value > 0.01 were regarded as failed. Any samples with >10% failed probes, and any probes with > 10% failure rate were removed from the dataset. Beta values from failed probes (approximately 0.001% of the dataset) were imputed using the *impute*.knn function as part of the *impute* R package.

Non-CpG probes (2,932), SNP-related probes as identified by Zhou et al.^33^ (82,108), and chrY probes were removed from the dataset. An additional 6,102 previously identified probes that followed a trimodal methylation pattern characteristic (unpublished data) of an underlying SNP were removed.

Background intensity correction and dye bias correction was performed using the *minfi* single sample preprocessNoob function. Probe bias correction was performed using the beta mixture quantile normalisation (BMIQ) algorithm.

The fraction of immune cell contamination, and the relative proportions of different immune cell subtypes in each sample, were estimated using the EpiDISH algorithm using the epithelial, fibroblast, and immune cell reference dataset. The top 1,000 most variable probes (ranked by standard deviation) were used in a principal component analysis. Statistical tests were performed in order to identify any anomalous associations between plate, sentrix position, date of array processing, date of DNA creation, study centre, immune contamination fraction, age, type (case versus control), and the top ten principal components. Finally, two-thirds of the discovery dataset was randomly selected for use as the training dataset and the remaining third was allocated to the internal validation dataset. This split was carried out once, and the same training and validation sets were used in all subsequent analyses.

A total of 113 samples were downloaded from the ENCODE database (https://www.encodeproject.org/; see Supplementary Table 6)^28^. The beta mixture quantile normalisation was applied to these samples after using *minfi* to extract beta values.

### Statistical analyses for classifier development

Contamination by immune cells presented a challenge with respect to the identification of differentially methylated positions (DMPs) as differential methylation that occurred solely in epithelial cells was diminished in samples with high IC and vice versa. In order to overcome this, we linearly regressed the beta values on IC for each CpG site, the linear models being fitted to cases and controls separately. The intercept points at IC = 0 were used as estimates of mean beta values in cases and controls in a pure epithelial cell population. The difference between these intercept points provided a delta-beta estimate in epithelial cells. The difference between intercept points at IC = 1 provided immune cell delta-beta estimates. An example is provided in Fig. 1c. A list of ranked CpGs was produced according to delta-beta estimates in epithelial cells.

The R package *glmnet* was used to train classifiers with a mixing parameter value of alpha = 0 (ridge penalty) and alpha = 1 (lasso penalty) with binomial response type. Data from the training dataset were used to fit the classifiers. The top n CpGs from the list of CpGs ranked by epithelial delta-beta estimates were used as inputs to the classifier. Ten-fold cross-validation was used inside the training set by the cv.*glmnet* function in order to determine the optimal value of the regularisation parameter lambda. The AUC was used as a metric of classifier performance which was evaluated on the internal validation dataset as a function of *n*, the number of CpGs used as inputs during training. The maximum value of *n* was 30,000.

The optimal classifier was selected based on the highest AUC obtained in the internal validation dataset. Once the optimal number of inputs was determined, the training and internal validation datasets were combined and the classifier was refitted using the entire discovery dataset with alpha and lambda fixed to their optimal values. This finalised classifier was then applied to the external validation dataset and the corresponding AUC was computed.

Denoting the top *n* CpGs as ${x}_{1},\ldots ,{x}_{n}$ and the regression coefficients from the trained classifier as ${w}_{1},\ldots ,{w}_{n}$ then WID-OC-index = $\mathop{\sum }\nolimits_{i=1}^{n}({w}_{i}{x}_{i}-\mu )/\sigma$ where $\mu$ and $\sigma$ are defined as the mean and standard deviation of the quantity $\mathop{\sum }\nolimits_{i=1}^{n}{w}_{i}{x}_{i}$ in the training dataset (i.e. the index is scaled to have zero mean and unit standard deviation in the training dataset). Code to compute the WID-OC-index is provided (DOI is 10.5281/zenodo.4757468, 2021; see also Code Availability section).

### Enrichment analyses

A gene set enrichment analysis (GSEA) was carried out by first selecting for each gene TSS200 region in the CpG with the largest epithelial delta-beta estimate (both hyper- and hypo-methylated). Genes were then ranked according to the absolute value of these delta-beta estimates. The C2 curated gene set, c2.all.v6.2.symbols.gmt, was downloaded from MSigDB. The *fgsea*R package was used to perform the enrichment analysis with parameters minSize, maxSize, and nperm set to 15, 500, and 10,000 respectively.

### Estimation of tumour DNA proportion

The EpiDISH algorithm provides an estimate of cell type proportions within a given sample. A reference dataset consisting of CpGs that are unique to each cell type must be provided. In order to construct such a reference dataset 11 epithelial, 7 fibroblast, 48 immune, and 11 high grade serous ovarian cancer cell line samples were downloaded from GEO (Supplementary Table 5). Each cell type was in turn compared to the other three cell types (which were combined into one group) in order to identify CpGs that are unique to that cell type. A Wilcoxon rank sum test was used to test for differential methylation at each CpG. For epithelial cells any CpGs with a *p*-value > 0.01 after false discovery rate (FDR) adjustment and an absolute difference in methylation > 0.54 were selected (204 in total). For fibroblasts any CpGs with FDR adjusted *p*-values > 0.01 and differential methylation >0.7 were selected (208 in total). For immune cells any CpGs with FDR adjusted *p*-values > 0.01 and differential methylation >0.89 were selected (225 in total). For high-grade serous ovarian carcinoma (HGSOC) cells, any CpGs with FDR adjusted *p*-values >0.01 and differential methylation >0.77 were selected (203 in total). The final reference dataset therefore consisted of 840 CpGs.

A similar protocol was followed for the endometrial cancer reference dataset using 9 endometrial cancer tissue samples. For epithelial cells any CpGs with a *p*-value > 0.01 after false discovery rate (FDR) adjustment and an absolute difference in methylation > 0.56 were selected (212 in total). For fibroblasts any CpGs with FDR adjusted *p*-values > 0.01 and differential methylation > 0.67 were selected (201 in total). For immune cells any CpGs with FDR adjusted *p*-values > 0.01 and differential methylation > 0.84 were selected (218 in total).

It was observed that the inferred proportion of tumour DNA and epithelial cells were strongly associated in control samples. Local polynomial regression fitting (using the loess R function) was used to regress the inferred tumour DNA proportion on the epithelial proportion (in control samples only, Supplementary Fig. 7b, c) and the residuals were used as estimates for tumour DNA proportion.

### Numerical simulation of tumour DNA contamination

Beta values from the single HGSOC cell line 1 sample (Fig. 4e) were combined with beta values from 144 cervical smear control samples with IC < 0.5 in the internal validation set. If ${\beta }_{t}\,$are beta values from the tumour cell line and ${\beta }_{c}$ are beta values from cervical samples and $\rho$ is the simulated proportion of tumour DNA then the simulated beta values are given by ${\beta =(1-\rho )\beta }_{c}+{\rho \beta }_{t}$. This was carried out  for all 144 control samples in order to simulate 144 cancer samples. The WID-OC-index was computed in both groups for different values of tumour proportion.

### Copy number variation (CNV) analysis

CNV analysis based on Illumina Methylation EPIC array data was conducted using the *conumee* R package (Version 1.22.0, Hovestadt & Zapatka)^34^, which was recently found to be the most reliable tool for EPIC array CNV calling^27^. Raw methylation data from HGSOC and normal fallopian tube samples was obtained from GEO (Accession no. GSE133556). Raw methylation data (MethylSet) from (a) the normal fallopian tube-HGSOC dataset and (b) cervical samples from controls and ovarian cancers was normalised using the SWAN function in *minfi*. CNVs were then called using *conumee*, whereby samples were initially normalised to samples processed in the same experiment with an assumed “flat” genome using multiple linear regression to control for probe and sample bias. Subsequently neighbouring probes were combined in a hybrid approach, resulting in predefined genomic bins, and segmented into regions of the same copy number state, which were visualised using the *conumee* CNV.genomeplot() function. To assess overall CNVs and CNV variability in distinct genomic bins, median or standard deviation per genomic bin compared to respective controls were computed for HGSOC samples and cervical samples from ovarian cancer cases and compared. For assessment of the association of CNV variability with inferred tumour DNA, the overall CNV standard deviation per sample was computed and plotted against inferred tDNA.

### Estimation of epithelial and immune variance

We aimed to estimate how much variability across the 14,000 CpGs in the WID-OC-index could be attributed to epithelial cells or immune cells. An example of a CpG with high variability in epithelial cells and low variability in immune cells is given in Supplementary Fig. 7d. For each CpG we applied the following model. We assumed that the epithelial beta values follow a beta distribution ${Beta}(\beta |{a}_{0},{b}_{0})$ with shape parameters ${a}_{0}\, > \, 0$ and ${b}_{0}\, > \, 0$, and that immune beta values followed ${Beta}(\beta |{a}_{1},{b}_{1})$ with shape parameters ${a}_{1}\, > \, 0$ and ${b}_{1}\, > \, 0$. We assumed that each sample is a combination of epithelial and immune cells and that ${\rho }_{i}\in [{{{{\mathrm{0,1}}}}}]$ is the proportion of immune cells in sample $i,{i}=1,\ldots ,N.$ The quantities ${\rho }_{i}$ were obtained from the EpiDISH algorithm. The following log likelihood function was numerically optimised with respect to ${a}_{0},{b}_{0},{a}_{1},{b}_{1}$:

$$L({a}_{0},{b}_{0},{a}_{1},{b}_{1})=-\frac{1}{N}\mathop{\sum }\limits_{i=1}^{N}\,\log \,[(1-{\rho }_{i})Beta({\beta }_{i}|{a}_{0},{b}_{0})+{\rho }_{i}Beta({\beta }_{i}|{a}_{1},{b}_{1})]$$

and the variance of the epithelial and immune beta distributions were used as estimates of epithelial and immune variance.

### SNP genotyping, QC and imputation

In total, 74 ovarian cancer case subjects and 225 controls from the methylation discovery cohort were genotyped using the Illumina 650k Infinium Global Screening Array (GSA). Whole blood DNA was normalised to 75 ng/µl and a total of 300 ng applied to the Infinium Global Screening Array – 24 V2 (Illumina, CA, USA) at UCL Genomics according to the manufacturer’s standard protocol.

One ovarian cancer case and one control subject from this cohort failed to genotype. Genotype calling was performed using GenomeStudio, with genetic variants found to be clustering poorly removed from further analyses. For duplicate genetic variant pairs, the variant within each pair with the lowest calling and clustering score was excluded. Autosomal SNPs were used in subsequent QC and PRS analyses (except for checks for sex mismatches, where the X chromosome was used to infer sex).

General subject and single nucleotide polymorphism (SNP) quality control (QC) was performed using PLINK version 1.9^35^. Four ovarian cancer cases and eight controls with a call rate less than 95% were excluded. Three controls were further removed due to genetically inferred sex not being female. Genetic variants with a missing genotype rate greater than 5%, minor allele frequency (MAF) less than 1% or a significant departure from Hardy–Weinberg equilibrium (*p*-value < 5 × 10^−6^) were excluded.

KING^36^, a relatedness inference algorithm, was used to identify duplicate/monozygotic twin or first-degree relative pairs. One control subject pair was identified as being a duplicate/monozygotic twin pair, and nine control pairs were inferred to be first-degree relatives. The subject within each related pair with the lowest call rate was excluded. After performing QC, 225 ovarian cancer case subjects, 816 controls, and 479,105 variants were retained in the SNP discovery sample.

Non-European subjects were identified by plotting the top two principal components, generated using GCTA version 1.26.0, for the SNP discovery samples and 270 HapMap phase II release 23 samples (CEU, YRI, JPT, and CHB individuals) downloaded in PLINK-formatted binary files. Subjects found not to cluster around HapMap European samples were excluded from further analyses. After excluding non-European subjects, 217 ovarian cancer cases and 752 controls were retained in the SNP discovery sample.

Using the Michigan Imputation Server^37^ and Haplotype Reference Consortium (HRC) reference panel, the SNP discovery dataset went through further QC before being phased (Eagle2) and imputed. Variants where strand, allele, genetic position or allele frequencies were not concordant with the HRC reference panel were removed before phasing and imputation using Strand Tools. After imputation, variants with imputation *R*^2^ < 0.5 were removed. LD-based clumping was performed to retain a set of independent variants (r^2^ > 0.1). 28 SNPs, associated with ovarian cancer, were used to develop an ovarian cancer polygenic risk score (PRS; Supplementary Table 4). We constructed an ovarian cancer PRS for each subject in the discovery cohort, such that the PRS is equal to:

$${{PRS}}_{j}=\mathop{\sum }\limits_{i=1}^{28}\hat{{\beta }_{i}}\,{x}_{{ij}}$$

where,$\,\hat{{\beta }_{i}}$ is the log odds ratio for the *i*-th SNP taken from publicly available ovarian cancer summary association results and ${x}_{{ij}}$ is the number of copies of the effect allele present in each discovery cohort subject. The ovarian cancer summary results used were based on weights given in Phelan et al.^38^, which were downloaded using GWAS Catalog^39^ (Accession number: GCST004415). Scores were generated using PLINK version 1.9^34^.

### Reporting summary

Further information on research design is available in the Nature Research Reporting Summary linked to this article.

## Supplementary information

Supplementary Information

Description of Supplementary Files

Supplementary Data 1

Reporting Summary

## Data Availability

The DNAme data generated in this study have been deposited in the European Genome-phenome Archive (EGA) database under accession codes EGAS00001005045 (cases) and EGAS00001005055 and are available under restricted access. SNP data for calculation of the polygenic risk score is also deposited for the above EGA studies, in EGA datasets EGAD00001008143 and EGAD00001008145 (cases and controls, respectively). Source data are provided with this paper. The remaining data are available within the Article and Supplementary Information. Source data are provided with this paper.

## Source data

Source Data

## Code availability

Software is available at github.com/IfWH-DoWC/OC-index.
