# Supplementary material for: The DNA methylome of cervical cells can predict the presence of ovarian cancer
Source: Nat Commun. 2022 Feb 1;13:448. doi: 10.1038/s41467-021-26615-y (PMC8807742; doi:10.1038/s41467-021-26615-y)
Supplement: Supplementary file 1 — Supplementary Information [file 41467_2021_26615_MOESM1_ESM.pdf]

SUPPLEMENTARY INFORMATION

The DNA methylome of cervical cells can predict the presence of ovarian cancer

James E. Barrett, Allison Jones, Iona Evans, Daniel Reisel, Chiara Herzog, Kantaraja Chindera, Mark Kristiansen, Olivia C. Leavy, Ranjit Manchanda, Line Bjørge, Michal Zikan, David Cibula and Martin Widschwendter

Inclusion criteria

Ovarian cancer:
- Morphology: high grade serous or endometrioid or mucinous or clear cell
- No treatment (surgery/chemotherapy) prior to sampling

Controls:
- Volunteers from general population
- Women attending hospital for benign women-specific conditions

Discovery Set (n=1111)			
		**Cervical DNAme**	**Matched SNP**
Training Set	Ovarian Cancer	159	
	Control	572	
Internal Validation	Ovarian Cancer	83	74
	Control	297	255
Total		1111	299

External Validation Set (n=272)		
		**Cervical DNAme**
External Validation	Ovarian Cancer	47
	Control	225
Total		272

Endometrial Cancer Set (n=514)	
	**Cervical DNAme**
Endometrial Cancer	217
Control	297
Total	514

Breast Cancer Set (n=626)	
	**Cervical DNAme**
Breast Cancer	329
Control	297
Total	626

BRCA Set (n=272)	
	**Cervical DNAme**
BRCA1	57
BRCA2	53
Control	114
Total	224

Supplementary Figure 1. Experimental design.

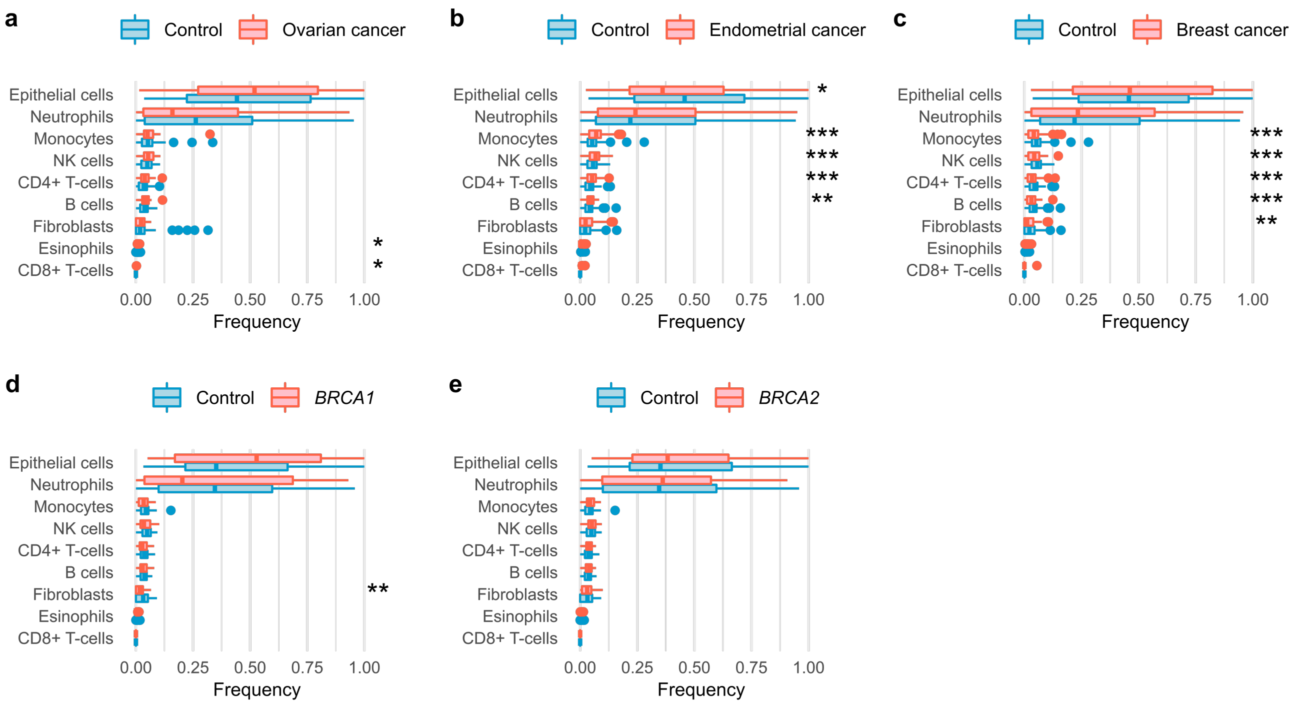

Supplementary Figure 2. Cell type distribution.
a Cell type distribution in the external validation set inferred using the EpiDISH algorithm. *p*-values were computed using a two-tailed Mann–Whitney test. For indicated significant differences, exact *p*-values = 0.03 (eosinophil), 0.03 (CD8+ T-cells). For box plots, the centre line of each box corresponds to the median. The lower and upper hinges correspond to the first and third quartiles. The upper whisker extends from the hinge to the largest value no further than 1.5 * IQR from the hinge (where IQR is the inter-quartile range). The lower whisker extends to the smallest value at most 1.5 * IQR of the hinge. Data beyond the end of the whiskers are plotted individually. **b** Cell type distribution in the endometrial cancer dataset inferred using the EpiDISH algorithm. Plot as in **a**. *p*-values were computed using a two-tailed Mann–Whitney test. For indicated significant differences, exact *p*-values = 0.029 (Epithelial), <0.001 (Monocytes), <0.001 (NK cells), <0.001 (CD4+ T-cells), 0.0014 (B cells). **c** cell type distribution in the breast cancer dataset inferred using the EpiDISH algorithm. Plot as in **a**. *p*-values were computed using a two-tailed Mann–Whitney test. For indicated significant differences, exact *p*-values = <0.001 (Monocytes), <0.001 (NK cells), <0.001 (CD4+ T-cells), <0.001 (B cells), 0.0076 (Fibroblast). **d** cell type distribution in the *BRCA1* dataset inferred using the EpiDISH algorithm. Plot as in **a**. *p*-values were computed using a two-tailed Mann–Whitney test. For indicated significant differences, exact *p*-values = 0.0028 (Fibroblast). **e** cell type distribution in the *BRCA2* dataset inferred using the EpiDISH algorithm. Plot as in **a**.

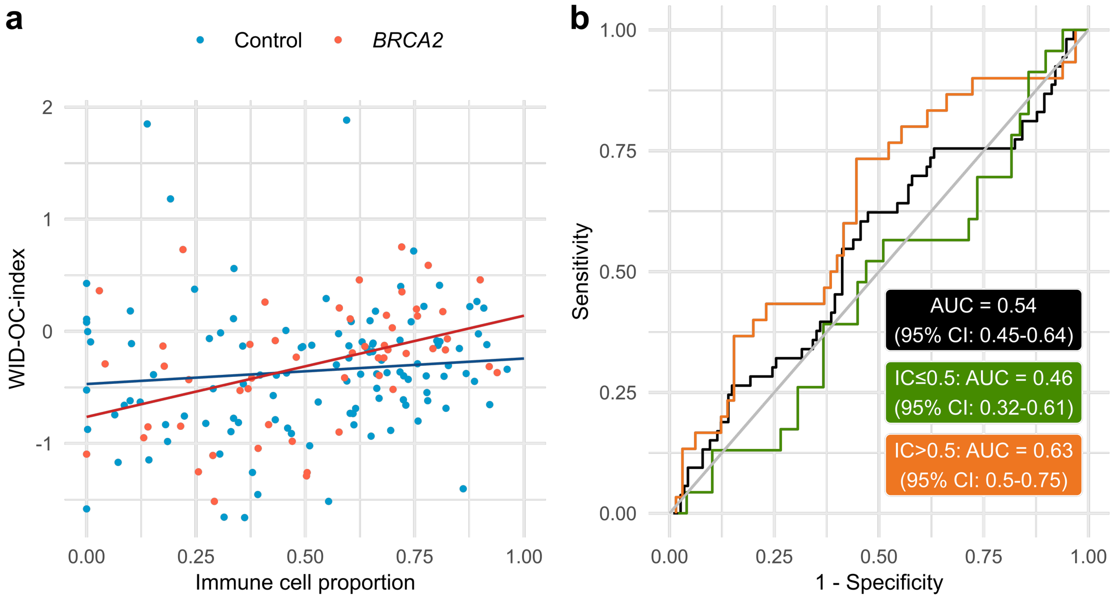

Supplementary Figure 3. The WID-OC-index in *BRCA2* mutation carriers.
a The WID-OC-index versus immune cell proportion in an independent cohort of *BRCA2* mutation carriers. **b** ROC curve from the *BRCA2* dataset.

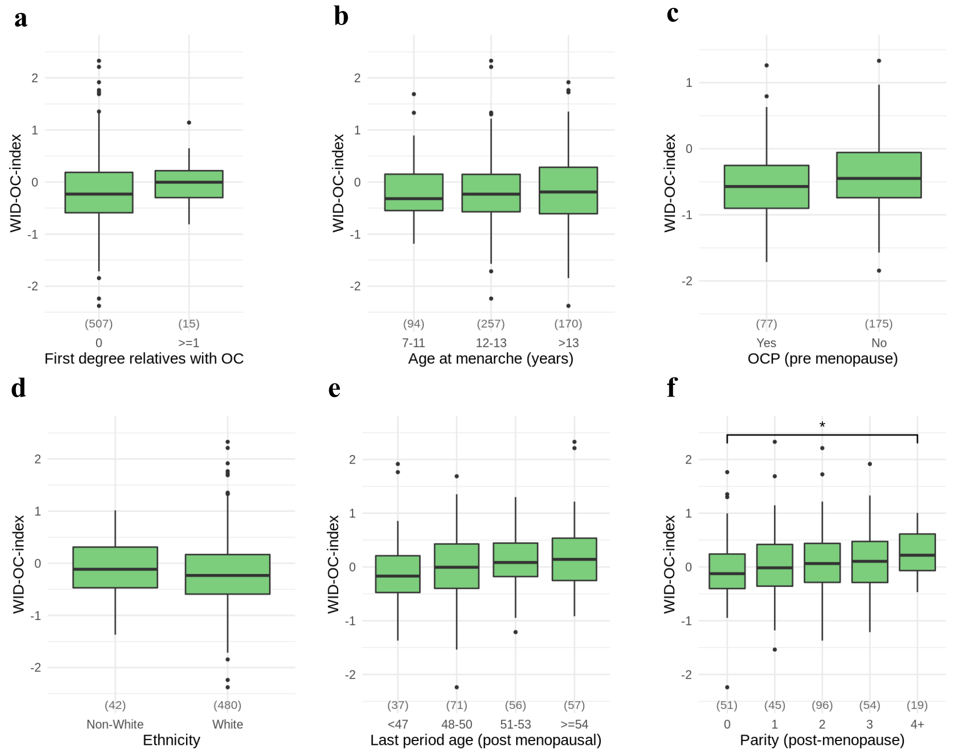

Supplementary Figure 4. The WID-OC-index and epidemiological factors.
Association of the WID-OC-index with **a** family history**, b** age at menarche, **c** oral contraceptive pill (OCP) use, **d** ethnicity, **e** last menstrual period, and **f** parity. *p*-values were computed using a two-tailed Mann–Whitney test. For indicated significant differences, exact *p*-values = 0.032. For box plots, the centre line of each box corresponds to the median. The lower and upper hinges correspond to the first and third quartiles. The upper whisker extends from the hinge to the largest value no further than 1.5 * IQR from the hinge (where IQR is the inter-quartile range). The lower whisker extends to the smallest value at most 1.5 * IQR of the hinge. Data beyond the end of the whiskers are plotted individually.

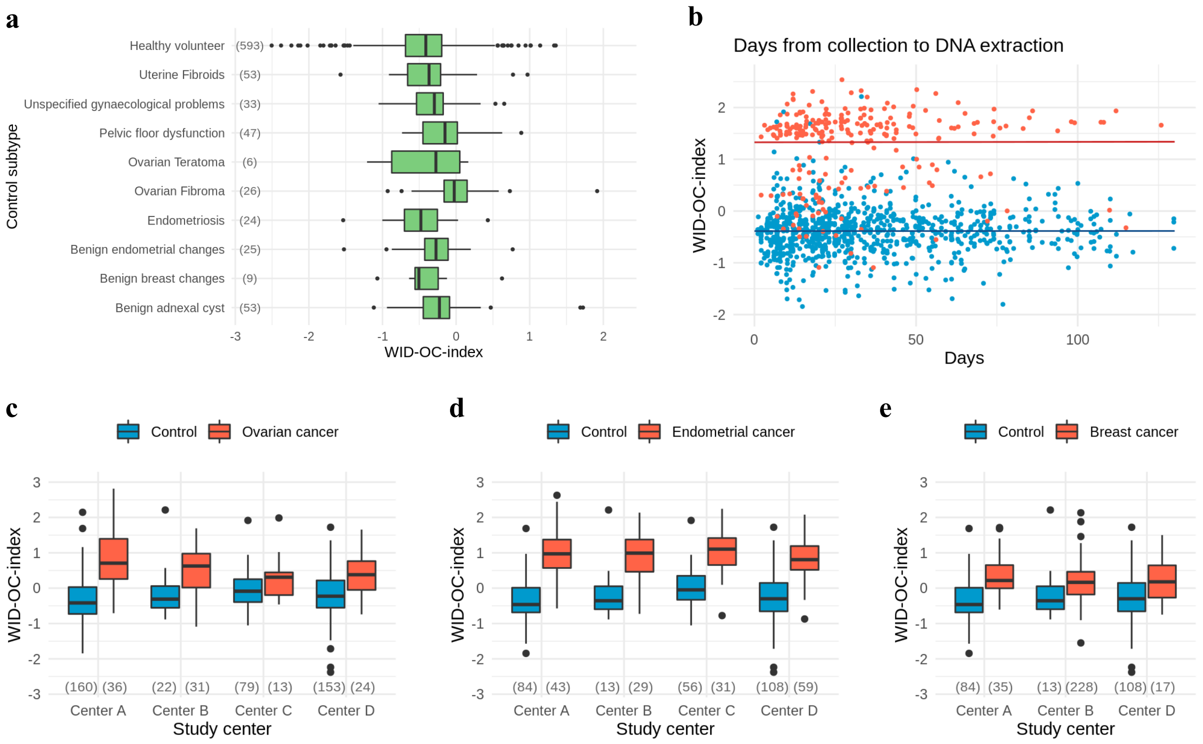

Supplementary Figure 5. The WID-OC-index in various control samples, as a function of storage time and collection centres.

a Distribution of the WID-OC-index among various subtypes of control samples. **b** Association between the WID-OC-index and time from sample collection to DNA extraction. **c** Distribution of the WID-OC-index across the four biggest study centers in the ovarian cancer internal and external validation sets. **d** Distribution of the WID-OC-index across the four biggest study centers in the endometrial cancer set. **e** Distribution of the WID-OC-index across the three biggest study centers in the breast cancer set. For box plots, the centre line of each box corresponds to the median. The lower and upper hinges correspond to the first and third quartiles. The upper whisker extends from the hinge to the largest value no further than 1.5 * IQR from the hinge (where IQR is the inter-quartile range). The lower whisker extends to the smallest value at most 1.5 * IQR of the hinge. Data beyond the end of the whiskers are plotted individually.

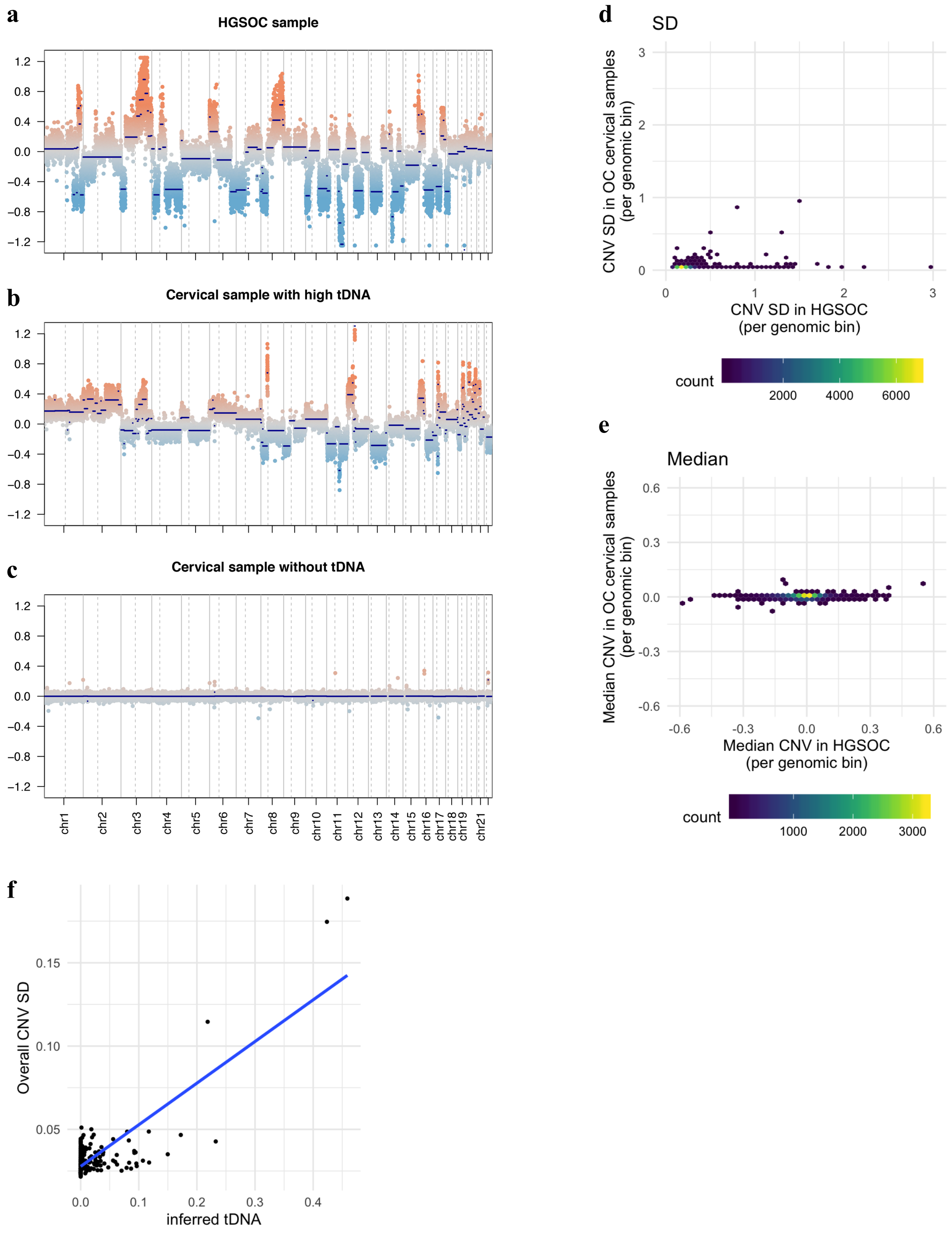

Supplementary Figure 6. Copy number variation in ovarian cancer tissue and cervical samples.

a Example genomic copy number variation (CNV) plot of a high grade serous ovarian carcinoma (HGSOC) sample. **b** Example of cervical sample from ovarian cancer patients with a high inferred tumour DNA fraction or **c** no tumour DNA. **d** and **e** median and standard deviation CNV per genomic bin in HGSOC and cervical samples. **f** CNV standard deviation per genomic bin versus inferred tumour DNA in cervical samples.

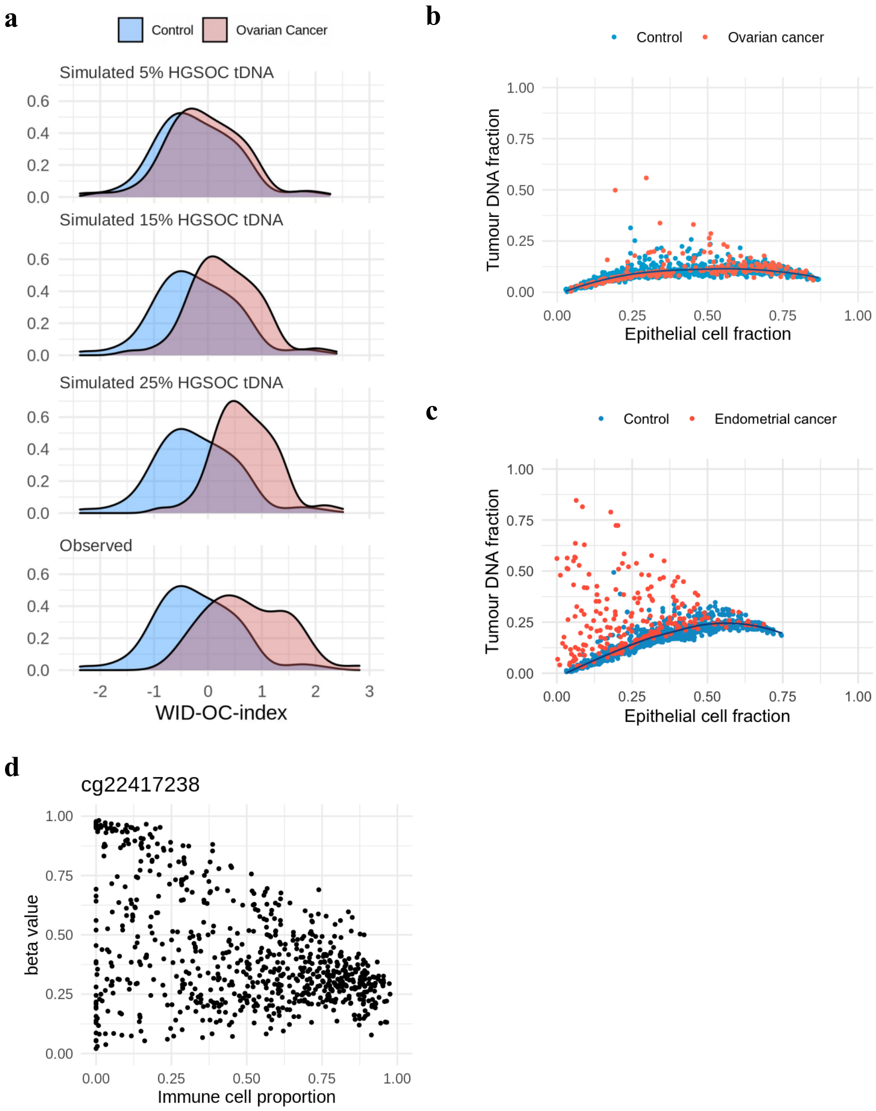

Supplementary Figure 7. The WID-OC-index is not driven by the presence of tumour DNA in the cervical sample.
a The distribution of the WID-OC-index based on numerically simulated mixtures of HGSOC tumour and cervical DNA. **b** The proportions of epithelial and tumour DNA in each sample inferred using the EpiDISH algorithm with a reference panel of epithelial, immune, fibroblast and tumour cell types in ovarian cancer and **c** endometrial cancer versus control. **d** An example of a CpG with a high epithelial variance and low immune cell variance.

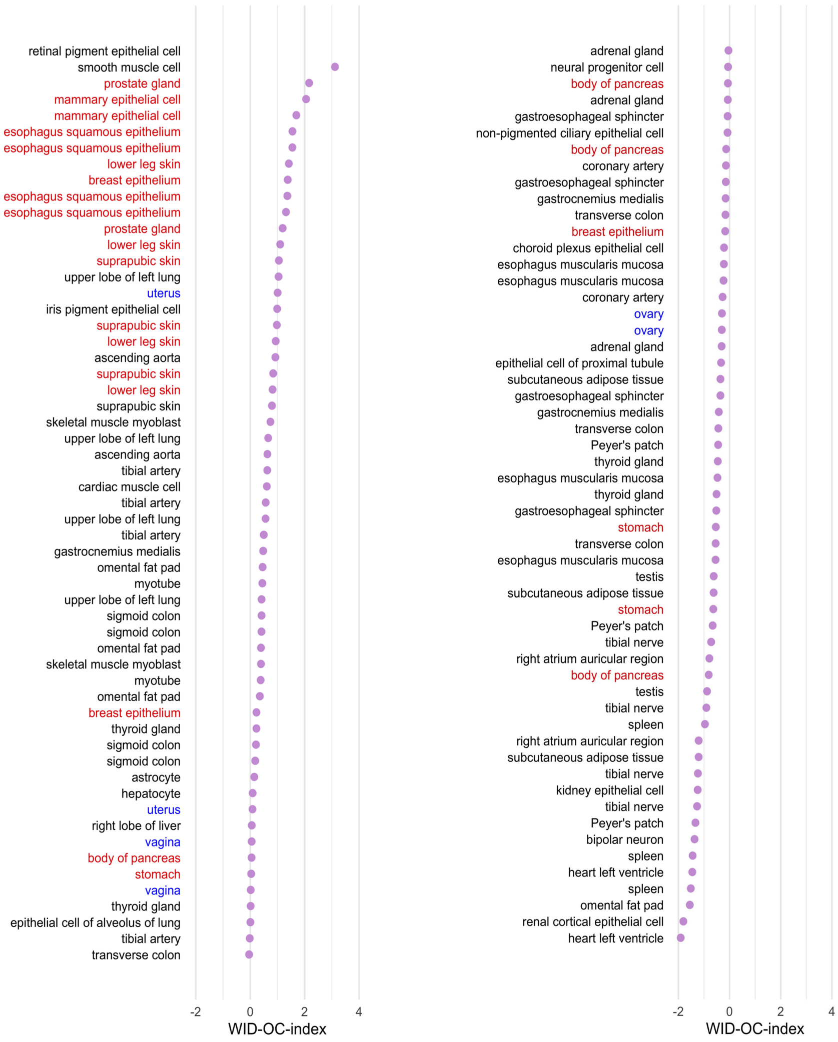

Supplementary Figure 8. The WID-OC-index evaluated in ENCODE tissue samples.
The WID-OC-index evaluated in ENCODE tissue samples. Tissue types at high risk of developing cancer in *BRCA* carriers are coloured red, low risk tissues are coloured blue.

A) Epidemiological characteristics

Discovery set

	Value	Control (N=869)	Cancer (N=242)	OR (95% CI)	P value
**Age (years)**	<52	538 (62%)	62 (26%)	1.00 (reference)	-
	52-64	208 (24%)	88 (36%)	3.66 (2.55,5.28)	$<10^{-11}$
	>64	123 (14%)	92 (38%)	6.47 (4.45,9.47)	$<10^{-16}$
**Age at menarche (years)**	<12	150 (17%)	51 (21%)	1.26 (0.83,1.89)	0.28
	12-13	437 (50%)	114 (47%)	0.96 (0.69,1.34)	0.82
	>13	277 (32%)	75 (31%)	1.00 (reference)	-
	Missing or Unknown	5 (1%)	2 (1%)	-	-
**Parous**	Yes	636 (73%)	187 (77%)	1.00 (reference)	-
	No	232 (27%)	53 (22%)	0.78 (0.55,1.09)	0.14
	Missing or Unknown	1 (0%)	2 (1%)	-	-
**Age at birth of first live child (years)**	<21	22 (3%)	12 (5%)	1.00 (reference)	-
	21-27	211 (24%)	106 (44%)	0.92 (0.44,1.99)	0.82
	>27	396 (46%)	67 (28%)	0.31 (0.15,0.68)	0.004
	Missing or Unknown	240 (28%)	57 (24%)	-	-
**Menopausal status**	Pre	509 (59%)	50 (21%)	1.00 (reference)	-
	Post	360 (41%)	192 (79%)	5.41 (3.88,7.66)	$<10^{-16}$
**Age at menopause (years)**	<46	423 (49%)	54 (22%)	0.35 (0.22,0.56)	$<10^{-4}$
	46-52	260 (30%)	111 (46%)	1.16 (0.75,1.82)	0.5
	>52	101 (12%)	37 (15%)	1.00 (reference)	-
	Missing or Unknown	85 (10%)	40 (17%)	-	-
**BMI (kg/m2)**	<25	445 (51%)	123 (51%)	1.00 (reference)	-
	25-30	275 (32%)	64 (26%)	0.84 (0.6,1.18)	0.32
	>30	149 (17%)	55 (23%)	1.34 (0.92,1.93)	0.13
**Oral contraceptive use (premenopausal)**	No	406 (80%)	43 (86%)	1.00 (reference)	-
	Yes	92 (18%)	5 (10%)	0.53 (0.18,1.26)	0.16
	Missing or Unknown	11 (2%)	2 (4%)	-	-
**Hormone replacement therapy (postmenopausal)**	No	306 (85%)	186 (97%)	1.00 (reference)	-
	Yes	50 (14%)	6 (3%)	0.2 (0.08,0.45)	$<10^{-4}$
	Missing or Unknown	4 (1%)	0 (0%)	-	-
**Smoking**	No	759 (87%)	200 (83%)	1.00 (reference)	-
	Yes	95 (11%)	39 (16%)	1.56 (1.03,2.32)	0.04
	Missing or Unknown	15 (2%)	3 (1%)	-	-
**First degree relatives with ovarian cancer**	0	823 (95%)	228 (94%)	1.00 (reference)	-
	1	46 (5%)	14 (6%)	1.11 (0.58,2)	0.75

External validation

	Value	Control (N=225)	Cancer (N=47)	OR (95% CI)	P value
**Age (years)**	<52	91 (40%)	15 (32%)	1.00 (reference)	-
	52-64	85 (38%)	15 (32%)	1.07 (0.49,2.35)	0.86
	>64	49 (22%)	17 (36%)	2.09 (0.96,4.62)	0.06
**Age at menarche (years)**	<12	34 (15%)	4 (9%)	0.62 (0.16,1.91)	0.42
	12-13	119 (53%)	29 (62%)	1.25 (0.62,2.59)	0.54
	>13	72 (32%)	14 (30%)	1.00 (reference)	-
**Parous**	Yes	166 (74%)	35 (74%)	1.00 (reference)	-
	No	59 (26%)	12 (26%)	0.97 (0.45,1.96)	0.94
**Age at birth of first live child (years)**	<21	16 (7%)	3 (6%)	1.00 (reference)	-
	21-27	61 (27%)	21 (45%)	1.76 (0.51,8.54)	0.39
	>27	84 (37%)	11 (23%)	0.68 (0.18,3.43)	0.61
	Missing or Unknown	64 (28%)	12 (26%)	-	-
**Menopausal status**	Pre	79 (35%)	12 (26%)	1.00 (reference)	-
	Post	146 (65%)	35 (74%)	1.56 (0.78,3.32)	0.21
**Age at menopause (years)**	<46	82 (36%)	12 (26%)	1.13 (0.41,3.5)	0.82
	46-52	91 (40%)	27 (57%)	2.27 (0.92,6.54)	0.08
	>52	47 (21%)	6 (13%)	1.00 (reference)	-
	Missing or Unknown	5 (2%)	2 (4%)	-	-
**BMI (kg/m2)**	<25	117 (52%)	28 (60%)	1.00 (reference)	-
	25-30	57 (25%)	13 (28%)	0.96 (0.45,1.97)	0.91
	>30	51 (23%)	6 (13%)	0.5 (0.18,1.22)	0.13
**Oral contraceptive use (premenopausal)**	No	28 (36%)	5 (42%)	1.00 (reference)	-
	Yes	50 (63%)	7 (58%)	0.78 (0.22,2.94)	0.7
	Missing or Unknown	1 (1%)	0 (0%)	-	-
**Hormone replacement therapy (postmenopausal)**	No	113 (77%)	30 (86%)	1.00 (reference)	-
	Yes	33 (23%)	5 (14%)	0.58 (0.18,1.53)	0.29
**Smoking**	No	190 (84%)	40 (85%)	1.00 (reference)	-
	Yes	34 (15%)	7 (15%)	0.99 (0.38,2.3)	0.99
	Missing or Unknown	1 (1%)	0 (0%)	-	-
**First degree relatives with ovarian cancer**	0	218 (97%)	46 (98%)	1.00 (reference)	-
	1	7 (3%)	1 (2%)	0.76 (0.03,4.53)	0.8

B) Clinical characteristics

		Discovery set	External validation set
	**Value**	**Cancer (N=242)**	**Cancer (N=47)**
**Age (years)**	<52	62 (26%)	15 (32%)
	52-64	88 (36%)	15 (32%)
	>64	92 (38%)	17 (36%)
**Menopausal status**	Pre	50 (21%)	12 (26%)
	Post	192 (79%)	35 (74%)
**Stage**	T1	51 (21%)	12 (26%)
	T2	31 (13%)	4 (9%)
	T3	126 (52%)	19 (40%)
	T4	32 (13%)	7 (15%)
	Missing or Unknown	2 (1%)	5 (11%)
**Grade**	I	34 (14%)	2 (4%)
	II	14 (6%)	2 (4%)
	III	187 (77%)	36 (77%)
	Missing or Unknown	7 (3%)	7 (15%)
**Histology**	High grade serous	168 (69%)	31 (66%)
	Low grade serous	20 (8%)	3 (6%)
	Mucinous	13 (5%)	3 (6%)
	Clear cell	19 (8%)	5 (11%)
	Endometrioid	17 (7%)	3 (6%)
	Other	5 (2%)	2 (4%)

Supplementary Table 1. Summary of (A) epidemiological and (B) clinical characteristics of the ovarian cancer discovery and validation set.
95% confidence intervals and p-values corresponding to odds ratios were computed using the median-unbiased estimation method.

List of significantly differentially methylated CpGs

CpG name	Chromosome	Position
cg04508482	chr4	996280
cg17805223	chr15	74002276
cg04361103	chr12	111127966
cg22268231	chr19	50922484
cg12569592	chr6	16753450
cg07451531	chr11	67040865
cg00858599	chr20	3220929
cg25330919	chr22	21934101
cg19869746	chr6	7107108
cg16254746	chr6	30698843
cg00163549	chr6	33246185
cg14062102	chr6	6932112
cg22251207	chr1	175287194
cg22596978	chr4	153329958
cg01292980	chr17	33378611
cg22188603	chr5	142243769
cg09083139	chr17	62637749
cg01125010	chr17	76134099
cg10395868	chr14	69263504
cg14885175	chr5	138720403
cg10513302	chr6	30698905
cg17355385	chr20	62368837
cg24424115	chr3	58476822
cg25399162	chr18	34824016
cg16872563	chr20	48247736
cg18440523	chr21	42690652
cg00656917	chr1	32422024
cg25598159	chr12	111127533
cg15824056	chr6	158402261
cg03142217	chr13	29315419
cg04380519	chr17	61778366
cg17445812	chr3	36986805
cg06607226	chr3	100617729
cg02856338	chr8	101822108
cg08828819	chr7	95025836
cg24101643	chr3	12667420
cg12057242	chr2	9603687
cg20318166	chr15	85360664
cg25507958	chr1	119532233
cg04741118	chr12	96720813
cg09926463	chr3	43680851

cg05424022	chr17	36665826
cg26689913	chr20	60822074
cg01473816	chr11	34535281
cg09387914	chr6	53187374
cg08435240	chr21	39643733
cg26461267	chr4	155662795
cg26364091	chr22	41633219
cg02305135	chr2	85361485
cg25310700	chr10	5702083
cg13232525	chr12	6432208
cg01147359	chr22	35880037
cg13053999	chr13	28549840
cg22974982	chr12	30323251
cg25701517	chr3	12671175
cg25496760	chr16	11051651
cg16915477	chr20	36339624
cg19009764	chr19	51588819
cg21082315	chr2	97474365
cg10398774	chr3	194117685
cg01026385	chr2	62442535
cg00856993	chr1	61953301
cg08790638	chr11	36476272
cg03949996	chr19	14586329
cg24126592	chr11	46389363
cg17714367	chr11	33555111
cg06088069	chr14	75895604
cg00340120	chr16	70616480
cg04770740	chr18	56236809
cg04483802	chr16	57006492
cg24440302	chr11	406829
cg24601412	chr7	101332409
cg11065467	chr10	133218425
cg20954977	chr2	232260116
cg04951962	chr1	167726178
cg15467877	chr1	36814475
cg02557933	chr2	220351040
cg06710195	chr1	108479452
cg13228355	chr20	53223230
cg13779162	chr11	69923033
cg08447387	chr8	131369596
cg13849515	chr17	54969261
cg09065876	chr17	45949756
cg25717182	chr11	76382149

cg06747543	chr19	18589894
cg09262559	chr12	12014507
cg21620075	chr1	207005028
cg15283622	chr2	191208022
cg23932491	chr10	119295056
cg18663382	chr6	30418789
cg13331354	chr19	18539841

Supplementary Table 2. List of differentially methylated CpGs that are significant after FDR adjustment.

	Age group	WID-OC index cut-off	Sensitivity (95% CI) [N/total]
**Specificity at 50%**	All ages	-0.23	83.0% (72.2-93.7) [39/47]
	< 50 years	-0.58	92.9% (79.4-100) [13/14]
	≥ 50 years	-0.04	84.8% (72.6-97.1) [28/33]
**Specificity at 75%**	All ages	0.22	61.7% (47.8-75.6) [29/47]
	< 50 years	-0.23	71.4% (47.8-95.1) [10/14]
	≥ 50 years	0.44	54.5% (37.6-71.5) [18/33]
**Specificity at 90%**	All ages	0.68	36.2% (22.4-49.9) [17/47]
	< 50 years	0.16	57.1% (31.2-83.1) [8/14]
	≥ 50 years	0.76	39.4% (22.7-56.1) [13/33]

Supplementary Table 3. Sensitivity of the WID-OC-index at different levels of specificity and age groups in the external validation set.

SNPs used to construct PRS

Chr	Position (build 37)	SNP name	Effect allele	Coefficients	Reference
12	121403724	12_121403724_G_A	A	-0.0603	Ref. 1
4	167187046	rs13113999	G	-0.03586	Ref. 1
8	129541931	rs1400482	A	-0.1629	Ref. 2
19	19528806	rs1469713	G	0.0352	Ref. 3
8	77320354	rs150293538	T	-0.133	Ref. 1
2	111896243	rs17041869	G	0.05732	Ref. 3
4	119949960	rs17329882	C	0.0724	Ref. 4
2	111818658	rs2165109	C	0.07511	Ref. 1
9	104943226	rs320203	A	-0.01177	Ref. 1
4	165908721	rs4691139	G	-0.001926	Ref. 5
9	106856793	rs4742903	C	0.05487	Ref. 3
19	17390291	rs4808075	C	0.1224	Ref. 6
1	22470407	rs56318008	T	0.06563	Ref. 4
1	38096421	rs58722170	C	0.07007	Ref. 4
22	28934313	rs6005807	C	0.1144	Ref. 1
3	156435640	rs62274041	A	0.3712	Ref. 6
2	177037311	rs711830	G	-0.1062	Ref. 7
17	46500673	rs7207826	C	0.1048	Ref. 6
17	36093022	rs7405776	G	-0.05083	Ref. 8
2	113972945	rs752590	G	0.02638	Ref. 7
8	82668818	rs76837345	G	0.1446	Ref. 9
5	1285974	rs7705526	A	0.08744	Ref. 8
10	105694301	rs7902587	T	0.06602	Ref. 1
11	61893972	rs7937840	T	0.04103	Ref. 3
15	91506637	rs8037137	C	-0.09911	Ref. 3
18	21405553	rs8098244	G	-0.03821	Ref. 1
3	190525516	rs9870207	G	-0.05224	Ref. 1
8	128817883	rs9886651	G	0.05831	Ref. 1

Supplementary Table 4. List of SNPs used to construct an ovarian cancer polygenic risk score. The coefficients are log odds ratios as provided by Phelan et. al. [1].

Samples used to construct EpiDISH reference panel

GEO accession number	Sample type
GSM999336	Epithelial
GSM999346	Epithelial
GSM999361	Epithelial
GSM999355	Epithelial
GSM999362	Epithelial
GSM999369	Epithelial
GSM999378	Epithelial
GSM999380	Epithelial
GSM999384	Epithelial
GSM999385	Epithelial
GSM999390	Epithelial
GSM999340	Fibroblast
GSM999342	Fibroblast
GSM999344	Fibroblast
GSM999345	Fibroblast
GSM999348	Fibroblast
GSM999350	Fibroblast
GSM999394	Fibroblast
GSM861653	Immune cell
GSM861654	Immune cell
GSM861655	Immune cell
GSM861656	Immune cell
GSM861657	Immune cell
GSM861658	Immune cell
GSM861659	Immune cell
GSM861660	Immune cell
GSM861661	Immune cell
GSM861662	Immune cell
GSM861663	Immune cell
GSM861664	Immune cell
GSM861665	Immune cell
GSM861666	Immune cell
GSM861667	Immune cell
GSM861668	Immune cell
GSM861669	Immune cell
GSM861670	Immune cell
GSM861671	Immune cell
GSM861672	Immune cell
GSM861673	Immune cell
GSM861674	Immune cell
GSM861675	Immune cell

GSM861676	Immune cell
GSM861677	Immune cell
GSM861678	Immune cell
GSM861679	Immune cell
GSM861680	Immune cell
GSM861681	Immune cell
GSM861682	Immune cell
GSM861683	Immune cell
GSM861684	Immune cell
GSM861685	Immune cell
GSM861686	Immune cell
GSM861687	Immune cell
GSM861688	Immune cell
GSM861689	Immune cell
GSM861690	Immune cell
GSM861691	Immune cell
GSM861692	Immune cell
GSM861693	Immune cell
GSM861694	Immune cell
GSM1836188	High grade serous ovarian cancer
GSM1836190	High grade serous ovarian cancer
GSM1836192	High grade serous ovarian cancer
GSM1836194	High grade serous ovarian cancer
GSM1836196	High grade serous ovarian cancer
GSM1836198	High grade serous ovarian cancer
GSM1836200	High grade serous ovarian cancer
GSM1836202	High grade serous ovarian cancer
GSM1836204	High grade serous ovarian cancer
GSM1836206	High grade serous ovarian cancer
GSM1836208	High grade serous ovarian cancer
ECHM1138	Endometrial cancer tissue sample
ECHM1158	Endometrial cancer tissue sample
ECHM1193	Endometrial cancer tissue sample
ECHM1768	Endometrial cancer tissue sample
ECHM1350	Endometrial cancer tissue sample
ECHM1359	Endometrial cancer tissue sample
ECHM1359	Endometrial cancer tissue sample
ECHM1503	Endometrial cancer tissue sample
ECHM1781	Endometrial cancer tissue sample

Supplementary Table 5. List of samples used to construct an EpiDISH reference panel for epithelial, fibroblast, immune, ovarian cancer, and endometrial cancer cells.

ENCODE samples used

Experiment accession	Biosample term name	Biosample type
ENCSR371REA	adrenal gland	tissue
ENCSR209XGZ	adrenal gland	tissue
ENCSR399KXO	adrenal gland	tissue
ENCSR846DDC	breast epithelium	tissue
ENCSR392LYN	breast epithelium	tissue
ENCSR393CCK	breast epithelium	in vitro differentiated cells
ENCSR148KKY	mammary epithelial cell	primary cell
ENCSR583ILE	mammary epithelial cell	primary cell
ENCSR976HYM	choroid plexus epithelial cell	tissue
ENCSR847BAX	astrocyte	primary cell
ENCSR079OXD	bipolar neuron	in vitro differentiated cells
ENCSR246VHI	neural progenitor cell	in vitro differentiated cells
ENCSR147FPX	sigmoid colon	tissue
ENCSR827WSS	sigmoid colon	tissue
ENCSR773EPM	sigmoid colon	tissue
ENCSR002EIR	sigmoid colon	tissue
ENCSR580LHO	transverse colon	tissue
ENCSR558ACF	transverse colon	tissue
ENCSR002LED	transverse colon	tissue
ENCSR090CRZ	transverse colon	tissue
ENCSR200LAH	esophagus squamous epithelium	tissue
ENCSR154ELD	esophagus squamous epithelium	tissue
ENCSR097SQO	esophagus squamous epithelium	tissue
ENCSR312XVJ	esophagus squamous epithelium	tissue
ENCSR096DBW	stomach	tissue
ENCSR340GPO	stomach	tissue
ENCSR899UFG	stomach	tissue
ENCSR675IVH	epithelial cell of proximal tubule	primary cell
ENCSR244HUE	kidney epithelial cell	primary cell
ENCSR963NNG	renal cortical epithelial cell	primary cell
ENCSR955LKF	hepatocyte	primary cell
ENCSR937LYZ	right lobe of liver	tissue
ENCSR422EPB	epithelial cell of alveolus of lung	primary cell
ENCSR113TRL	upper lobe of left lung	tissue
ENCSR493EGV	upper lobe of left lung	tissue
ENCSR426CDE	upper lobe of left lung	tissue
ENCSR444YPR	upper lobe of left lung	tissue
ENCSR705PDD	body of pancreas	tissue
ENCSR597BUD	body of pancreas	tissue
ENCSR940ZHS	body of pancreas	tissue
ENCSR922EBK	body of pancreas	tissue

ENCSR080HYX	prostate gland	tissue
ENCSR415PYP	prostate gland	tissue
ENCSR744AJV	ovary	tissue
ENCSR511SNB	ovary	tissue
ENCSR942OLI	testis	tissue
ENCSR304AIL	testis	tissue
ENCSR604PTS	lower leg skin	tissue
ENCSR203HAK	lower leg skin	tissue
ENCSR301SLO	lower leg skin	tissue
ENCSR461NFO	lower leg skin	tissue
ENCSR575WOH	suprapubic skin	tissue
ENCSR448FCV	suprapubic skin	tissue
ENCSR353IUV	suprapubic skin	tissue
ENCSR792ATG	suprapubic skin	tissue
ENCSR809OPY	myotube	primary cell
ENCSR468IFF	myotube	in vitro differentiated cells
ENCSR738XQU	skeletal muscle myoblast	primary cell
ENCSR343SAU	skeletal muscle myoblast	primary cell
ENCSR420WUN	smooth muscle cell	in vitro differentiated cells
ENCSR001NCN	thyroid gland	tissue
ENCSR329WAK	thyroid gland	tissue
ENCSR173NTZ	thyroid gland	tissue
ENCSR406QEF	thyroid gland	tissue
ENCSR803DDS	uterus	tissue
ENCSR889TZA	uterus	tissue
ENCSR394PUR	vagina	tissue
ENCSR190WYF	vagina	tissue
ENCSR731SPT	ascending aorta	tissue
ENCSR486SMB	ascending aorta	tissue
ENCSR449VMS	cardiac muscle cell	tissue
ENCSR582BMR	coronary artery	tissue
ENCSR688OHW	coronary artery	tissue
ENCSR472PKR	esophagus muscularis mucosa	tissue
ENCSR822VTU	esophagus muscularis mucosa	tissue
ENCSR701SVQ	esophagus muscularis mucosa	tissue
ENCSR871SFO	esophagus muscularis mucosa	tissue
ENCSR248EIV	gastrocnemius medialis	tissue
ENCSR069UIN	gastrocnemius medialis	tissue
ENCSR193BIR	gastrocnemius medialis	tissue
ENCSR262IUB	gastroesophageal sphincter	tissue
ENCSR598BUX	gastroesophageal sphincter	tissue
ENCSR905RZU	gastroesophageal sphincter	tissue
ENCSR215SBD	gastroesophageal sphincter	tissue

ENCSR515ZCU	heart left ventricle	tissue
ENCSR190PQG	heart left ventricle	tissue
ENCSR754ANZ	iris pigment epithelial cell	primary cell
ENCSR528NFI	non-pigmented ciliary epithelial cell	primary cell
ENCSR306JCS	omental fat pad	tissue
ENCSR995PGW	omental fat pad	tissue
ENCSR733WXF	omental fat pad	tissue
ENCSR662NBA	omental fat pad	tissue
ENCSR467AVQ	Peyer's patch	tissue
ENCSR719GFJ	Peyer's patch	tissue
ENCSR201NNA	Peyer's patch	tissue
ENCSR899LHQ	retinal pigment epithelial cell	primary cell
ENCSR517JQA	right atrium auricular region	tissue
ENCSR280LMY	right atrium auricular region	tissue
ENCSR756BTI	spleen	tissue
ENCSR276YFP	spleen	tissue
ENCSR584HJL	spleen	tissue
ENCSR733HHJ	subcutaneous adipose tissue	tissue
ENCSR315CVG	subcutaneous adipose tissue	in vitro differentiated cells
ENCSR418YFM	subcutaneous adipose tissue	tissue
ENCSR962JMK	subcutaneous adipose tissue	tissue
ENCSR991SII	tibial artery	tissue
ENCSR425TKT	tibial artery	tissue
ENCSR646XKN	tibial artery	tissue
ENCSR050XGE	tibial artery	tissue
ENCSR729VBL	tibial nerve	tissue
ENCSR061NRX	tibial nerve	tissue
ENCSR551DKY	tibial nerve	tissue
ENCSR039CGW	tibial nerve	tissue

Supplementary Table 6. List of ENCODE samples used.

A) GSEA (hyper-methylated)

pathway	pval	padj	ES	NES	brief_description
WEBER METHYLATED HCP IN SPERM UP	0.0003	0.1588164	0.84871664	1.61	Methylated germline-specific genes with high-CpG-density promoters (HCP) in sperm.
WEBER METHYLATED HCP IN FIBROBLAST DN	0.0001	0.08739288	0.80776345	1.6	Unmethylated germline-specific genes with high-CpG-density promoters (HCP) in primary fibroblasts.
BIOCARTA IGF1R PATHWAY	0.0050	0.59251466	0.76941079	1.47	Multiple antiapoptotic pathways from IGF-1R signaling lead to BAD phosphorylation
WEBER METHYLATED HCP IN SPERM DN	0.0034	0.44735944	0.76075071	1.46	Unmethylated germline-specific genes with high-CpG-density promoters (HCP) in sperm.
WANG BARRETTS ESOPHAGUS AND ESOPHAGUS CANCER DN	0.0017	0.29728449	0.74190718	1.46	Genes down-regulated in esophageal adenocarcinoma (EAC) and Barret's esophagus (BE) relative to normal esophagi.
MURATA VIRULENCE OF H PILORI	0.0065	0.60604565	0.75359184	1.45	Selected genes up-regulated in WT-A10 cells (gastric epithelium) expressing the H. pilori virulence gene CagA.
JI METASTASIS REPRESSED BY STK11	0.0082	0.65271476	0.72704432	1.41	Adenocarcinoma metastatic program genes up-regulated in A549 and H2126 cells (lung cancer) lacking functional STK11 [GeneID=6794] but down-regulated by the normal gene.
BIOCARTA BAD PATHWAY	0.0134	0.85364139	0.72817675	1.4	Regulation of BAD phosphorylation
REACTOME INCRETIN SYNTHESIS SECRETION AND INACTIVATION	0.0222	1	0.73553555	1.39	Genes involved in Incretin Synthesis, Secretion, and Inactivation
REACTOME NUCLEOTIDE BINDING DOMAIN LEUCINE RICH REPEAT CONTAINING RECEPTOR NLR SIGNALING PATHWAYS	0.0071	0.60604565	0.70309409	1.39	Genes involved in Nucleotide-binding domain, leucine rich repeat containing receptor (NLR) signaling pathways
BIOCARTA EDG1 PATHWAY	0.0193	0.97242147	0.72031713	1.38	Phospholipids as signalling intermediaries
AMIT SERUM RESPONSE 480 MCF10A	0.0143	0.8948938	0.71226142	1.38	Genes whose expression peaked at 480 min after stimulation of MCF10A cells with serum.
BIOCARTA IGF1 PATHWAY	0.0251	1	0.7253061	1.38	IGF-1 Signaling Pathway
PEPPER CHRONIC LYMPHOCYTIC LEUKEMIA DN	0.0253	1	0.72497831	1.38	Genes down-regulated in CD38+ [GeneID=952] CLL (chronic lymphocytic leukemia) cells.
MINGUEZ LIVER CANCER VASCULAR INVASION DN	0.0274	1	0.7227135	1.38	Genes under-expressed in hepatocellular carcinoma (HCC) with vascular invasion.
REACTOME AQUAPORIN MEDIATED TRANSPORT	0.0055	0.59470591	0.6762393	1.35	Genes involved in Aquaporin-mediated transport
LEE LIVER CANCER	0.0154	0.91900421	0.68623303	1.35	Genes down-regulated in tumor compared to non-tumor liver samples from patients with hepatocellular carcinoma (HCC).
RICKMAN HEAD AND NECK CANCER C	0.0008	0.23295448	0.65586807	1.35	Cluster c: genes identifying an intrinsic group in head and neck squamous cell carcinoma (HNSCC).
VALK AML WITH EVI1	0.0381	1	0.70228942	1.34	Genes that best predicted acute myeloid leukemia (AML) with the up-regulated expression of EVI1 [GeneID=2122].
HEDENFALK BREAST CANCER BRACX UP	0.0471	1	0.70606063	1.34	Up-regulated genes distinguishing between two groups of non-BRCA1/BRCA2 [GeneID=672;675] breast tumors (BRACx): group A vs group B.

B) GSEA (hypo-methylated)

pathway	pval	padj	ES	NES	brief_description
REACTOME DEFENSINS	0.0013	0.10015572	0.78789775	1.57	Genes involved in Defensins
KEGG PHOSPHATIDYLINOSITOL SIGNALING SYSTEM	0.0015	0.10226177	0.7599521	1.54	Phosphatidylinositol signaling system
KEGG FC EPSILON RI SIGNALING PATHWAY	0.0007	0.08614953	0.73311197	1.53	Fc epsilon RI signaling pathway
REACTOME CYTOCHROME P450 ARRANGED BY SUBSTRATE TYPE	0.0024	0.12075567	0.75740656	1.52	Genes involved in Cytochrome P450 - arranged by substrate type
KORKOLA EMBRYONAL CARCINOMA UP	0.0038	0.15655647	0.74991	1.5	Genes from the 12p region that were up-regulated in embryonic carcinoma tumors compared to normal testis.
SETLUR PROSTATE CANCER TMPRSS2 ERG FUSION UP	0.0012	0.10015572	0.7223705	1.5	Genes up-regulated in prostate cancer samples bearing the fusion of TMPRSS2 with ERG [GeneID=7113;2078].
PID CXCR4 PATHWAY	0.0010	0.09992496	0.71404745	1.5	CXCR4-mediated signaling events
WANG BARRETTS ESOPHAGUS UP	0.0040	0.15708054	0.73810972	1.49	Genes up-regulated in Barrett's esophagus compared to the normal tissue.
DUNNE TARGETS OF AML1 MTG8 FUSION UP	0.0039	0.15655647	0.72975401	1.48	Genes up-regulated in Kasumi-1 cells (acute myeloid leukaemia (AML) with the t(8;21) translocation) after knockdown of the AML1 MTG8 fusion [GeneID=861;862] by RNAi.
ROSS AML WITH AML1 ETO FUSION	0.0032	0.142429	0.72128583	1.48	Top 100 probe sets for pediatric acute myeloid leukemia (AML) subtype t(8;21) ; has AML1 ETO fusion [GeneID=861;862].
NAKAYAMA SOFT TISSUE TUMORS PCA1 UP	0.0031	0.14186343	0.71766274	1.48	Top 100 probe sets contrubuting to the positive side of the 1st principal component; predominantly associated with spindle cell and pleomorphic sarcoma samples.
ELVIDGE HYPOXIA BY DMOG DN	0.0092	0.21617838	0.72362594	1.45	Genes down-regulated in MCF7 cells (breast cancer) treated with hypoxia mimetic DMOG [PubChem=3080614].
SENESE HDAC2 TARGETS UP	0.0021	0.11188238	0.68681216	1.45	Genes up-regulated in U2OS cells (osteosarcoma) upon knockdown of HDAC2 [GeneID=3066] by RNAi.
TURASHVILI BREAST DUCTAL CARCINOMA VS LOBULAR NORMAL DN	0.0023	0.11859687	0.68869002	1.45	Genes down-regulated in ductal carcinoma vs normal lobular breast cells.
PID TCR PATHWAY	0.0042	0.15997597	0.69586702	1.45	TCR signaling in naïve CD4+ T cells
MCLACHLAN DENTAL CARIES UP	0.0002	0.05326134	0.6449647	1.43	Genes up-regulated in pulpal tissue extracted from carious teeth.
PID TCR CALCIUM PATHWAY	0.0146	0.2928882	0.71547883	1.42	Calcium signaling in the CD4+ TCR pathway
OLSSON E2F3 TARGETS UP	0.0120	0.26495346	0.6992975	1.41	Genes up-regulated in the 5637 cell line (bladder cancer) after knockdown of E2F3 [GeneID=1871] by RNAi.
REACTOME SIGNALING BY SCF KIT	0.0102	0.23677066	0.68438925	1.41	Genes involved in Signaling by SCF-KIT
FLECHNER BIOPSY KIDNEY TRANSPLANT REJECTED VS OK UP	0.0060	0.17764438	0.6703189	1.4	Genes up-regulated in kidney biopsies from patients with acute transplant rejection compared to the biopsies from patients with well functioning kidneys more than 1-year post transplant.

Supplementary Table 7. GSEA top enriched pathways. (A) The top twenty enriched gene pathways based on hyper- methylated CpGs located in TSS200 regions. (B) The top twenty pathways on hypo- methylated CpGs. Pathways have been ranked by the normalized enrichment scores (NES).

Name	Sequence
ZNF154 Forward Primer 5’ – 3’	TTTATTTTAGGTTTGACGTGGGTTT
ZNF154 Reverse Primer 5’ – 3’	CGTCGTCCCTCCTACACGAA
ZNF154 Probe 5' 6-FAM - 3' BHQ-1	TAGGGCGGCGTCGTTAAGGTTTAGACG
COL2A1 Forward Primer 5’ – 3’	GGGAAGATGGGATAGAAGGGAATAT
COL2A1 Reverse Primer 5’ – 3’	TCTAACAATTATAAACTCCAACCACCAA
COL2A1 Probe 5' 6-FAM - 3' BHQ-1	CCTTCATTCTAACCCAATACCTATCCCACCTCTAAA

Supplementary Table 8. List of primer and probe sequences for ZNF154 detection.

AUC	Area under the curve
DNAme	DNA methylation
ENCODE	Encyclopedia of DNA Elements
EPIDISH	Hierarchical Epigenetic Dissection of Intra-Sample-Heterogeneity
HGSOC	High grade serous ovarian cancer
ROC	Receiver operating characteristic
WID-OC-index	Women's risk IDentification for Ovarian Cancer index
SNP	Single nucleotide polymorphism
PRS	Polygenic risk score

Supplementary Table 9. List of abbreviations.

Supplementary references

[1] Phelan, Catherine M., et al. "Identification of 12 new susceptibility loci for different histotypes of epithelial ovarian cancer." Nature genetics 49.5 (2017): 680.

[2] Earp, Madalene, et al. "A targeted genetic association study of epithelial ovarian cancer susceptibility." Oncotarget 7.7 (2016): 7381.

[3] Kar, Siddhartha P., et al. "Genome-wide meta-analyses of breast, ovarian, and prostate cancer association studies identify multiple new susceptibility loci shared by at least two cancer types." Cancer discovery 6.9 (2016): 1052-1067.

[4] Kuchenbaecker, Karoline B., et al. "Identification of six new susceptibility loci for invasive epithelial ovarian cancer." Nature genetics 47.2 (2015): 164.

[5] Couch, Fergus J., et al. "Genome-wide association study in BRCA1 mutation carriers identifies novel loci associated with breast and ovarian cancer risk." PLoS Genet 9.3 (2013): e1003212.

[6] Pharoah, Paul DP, et al. "GWAS meta-analysis and replication identifies three new susceptibility loci for ovarian cancer." Nature genetics 45.4 (2013): 362-370.

[7] Kelemen, Linda E., et al. "Genome-wide significant risk associations for mucinous ovarian carcinoma." Nature genetics 47.8 (2015): 888.

[8] Shen, Hui, et al. "Epigenetic analysis leads to identification of HNF1B as a subtype-specific susceptibility gene for ovarian cancer." Nature communications 4.1 (2013): 1-10.

[9] Lee, Alice W., et al. "A splicing variant of TERT identified by GWAS interacts with menopausal estrogen therapy in risk of ovarian cancer." International journal of cancer 139.12 (2016): 2646-2654.
