# Supplementary material for: The DNA methylome of cervical cells can predict the presence of ovarian cancer
Source: Nat Commun. 2022 Feb 1;13:448. doi: 10.1038/s41467-021-26615-y (PMC8807742; doi:10.1038/s41467-021-26615-y)
Supplement: Supplementary file 2 — Description of Supplementary Files [file 41467_2021_26615_MOESM2_ESM.docx]

**Description of Additional Supplementary Files**

**The DNA methylome of cervical cells can predict risk of ovarian cancer**

James E. Barrett, Allison Jones, Iona Evans, Daniel Reisel, Chiara Herzog, Kantaraja Chindera, Olivia C Leavy, Mark Kristiansen, Ranjit Manchanda, Line Bjørge, Michal Zikan, David Cibula and Martin Widschwendter

File Name: **Supplementary Data 1**

Description: The 14.000 CpGs comprising the WID-OC-index and their regression coefficients
